# Individual and team profiling to support theory of mind in artificial social intelligence

**DOI:** 10.1038/s41598-024-63122-8

**Published:** 2024-06-02

**Authors:** Rhyse Bendell, Jessica Williams, Stephen M. Fiore, Florian Jentsch

**Affiliations:** 1 Team Performance Laboratory, Institute for Simulation and Training, University of Central Florida, Orlando, FL 32816 USA; 2 Cognitive Sciences Laboratory, Institute for Simulation and Training, University of Central Florida, Orlando, FL 32816 USA; 3 Department of Philosophy, University of Central Florida, Orlando, FL 32816 USA; 4 Department of Psychology, University of Central Florida, Orlando, FL 32816 USA; 5 School of Modeling, Simulation, and Training, University of Central Florida, Orlando, FL 32816 USA

**Keywords:** Human behaviour, Psychology

## Abstract

We describe an approach aimed at helping artificial intelligence develop theory of mind of their human teammates to support team interactions. We show how this can be supported through the provision of quantifiable, machine-readable, a priori information about the human team members to an agent. We first show how our profiling approach can capture individual team member characteristic profiles that can be constructed from sparse data and provided to agents to support the development of artificial theory of mind. We then show how it captures features of team composition that may influence team performance. We document this through an experiment examining factors influencing the performance of ad-hoc teams executing a complex team coordination task when paired with an artificial social intelligence (ASI) teammate. We report the relationship between the individual and team characteristics and measures related to task performance and self-reported perceptions of the ASI. The results show that individual and emergent team profiles were able to characterize features of the team that predicted behavior and explain differences in perceptions of ASI. Further, the features of these profiles may interact differently when teams work with human versus ASI advisors. Most strikingly, our analyses showed that ASI advisors had a strong positive impact on low potential teams such that they improved the performance of those teams across mission outcome measures. We discuss these findings in the context of developing intelligent technologies capable of social cognition and engage in collaborative behaviors that improve team effectiveness.

## Introduction

The role of human-autonomy teams (HAT;^1^) is expanding rapidly in numerous industries, including healthcare, transportation, military operations, space exploration, and manufacturing^2^. Artificially intelligent (AI) autonomous agents excel in many forms of taskwork^3,4^ and are advancing to the point that they are nearly able to function as teammates in support of some tasks. But these advances have not been made in what would be considered teamwork. In the organizational sciences, a fundamental distinction is made between teamwork and taskwork. Taskwork is used to describe the form of work needing to be accomplished by a team (e.g., develop a new product; rescue victims from a burning building). Teamwork is used to describe the collaborative processes they need to execute to achieve their objectives (e.g., engage in leadership behaviors, communicate effectively, manage conflict, etc.). Thus, for AI to be capable of teamwork, it needs to understand the attitudes, behaviors, and cognition humans rely on during collaboration^1,5^.

Effective social interactions and collaboration in human-agent teaming depends on the development of social intelligence in AI agents whereby agents possess the necessary tools to monitor and engage in the exchange of social information^6,7^. This means success relies on the ability of autonomous agents to demonstrate social-cognitive capabilities to collaborate effectively with their human counterparts. More specifically, an ASI agent would have the capability to encode and decode socially communicative information contained within social signals. This requires an agent to perceive social cues (e.g., words spoken, intonation, body language, facial expression, etc.), and accurately interpret meaning from them, then to produce social signals to convey the intended social information^6,8^. This is an ongoing, interdisciplinary research challenge cutting across disciplines like computer science, engineering, and psychology requiring advances in computer vision, natural language processing, coupled with theory and methods in the social sciences^9^. Although AI has made substantial progress in more general interactions thanks to advancements in large language models, they remain largely socially ignorant. Until recently, they were unable to learn about a user and adapt their outputs over the course of an interaction. Further, they do not retain these memories or the information they learned in subsequent interaction, even with the same user. Such capabilities are essential for AI to demonstrate the kinds of social-cognitive capabilities foundational to interpersonal interactions.

Part of the difficulty in developing an ASI that is capable of engaging in effective social interactions and collaboration with humans is the need for social intelligence that is comparable to humans. Human-centered AI is increasingly recognized as an important area for improving collaborations between humans and machines^10^. Within that space, a crucial component of human-centered AI focuses on the social-cognitive aspects of human intelligence. Through the use of what is typically referred to as Theory of Mind (ToM), a core element of social cognition, humans develop their abilities to make inferences about situations and adapt the way they communicate with consideration to the situational context, such as if they are speaking to a novice or an expert in the topic, or when they’ve determined an individual has false or incomplete beliefs^11^. ToM is the ability to make these mental state attributions, including inferences and predictions about another, in the course of observing their actions, or during interaction. ToM processes enable us to understand and infer what another is thinking, feeling, and doing, and to interact with others based upon these mental state attributions.

### Artificial theory of mind

For artificial, socially intelligent systems to truly be able to effectively engage in social interactions with their human counterparts, they need to be able to infer, interpret, simulate, and predict human mental states, beliefs, and knowledge. To be able to make such mental state attributions, AI must be imbued with an Artificial Theory of Mind (AToM;^12^). By adapting models of human ToM to serve as an analog for an Artificial Theory of Mind (AToM), an agent could utilize these models to determine how to best communicate information in a way that is appropriate considering certain characteristics or features, such as prior experience or existing knowledge, the context of a situation, prior interactions, and skills or capabilities^12^, a skill that humans learn through interactions they have over the course of their development. If an agent can either be provided with a priori data (prior to interacting with the human) or learn about a particular human or team through human-agent interactions, an ASI can calibrate their internal models by taking into account existing knowledge of their human counterpart. Further, ASI can more effectively communicate in human-understandable ways, as humans are able to do, by providing clarification and transparency to their decision-making processes and the factors that influence them^13^. Research has shown that increasing transparency in communication and explanations of ASI decision-making fosters trust, finding that the humans understanding of the AI’s behavior and the predictability of the AI’s actions both impact the trust a human places in the AI^14^. The capability for an AI to adjust its outputs automatically to reconcile misunderstandings or false beliefs in the human counterpart can be facilitated by AToM processes and can be done in ways analogous to how humans use ToM^12^.

Theory of mind in humans is improved through interactions and explanations given by others, which allows humans to develop sensitivity to certain cues in their environment (which may differ across cultures, regions, or background^6^). This sensitivity, based on countless experiences, allows humans to perceive nuanced, situated meanings contained within social interactions, to infer another’s mental state based on observable behavior, and to predict future behavior^15^. An agent could be trained through interactions if they are able to maintain a base model that integrates prior experiences for use in present contexts and iterates over these models over time to become more accurate and interact more effectively^12^. However, traditional intelligent systems typically are not provided with the information they need to develop meaningful, iteratively updated models of their human counterparts. Further, they do not have the capability to extend those conceptualizations to more complex second-order attributions interrelating teammates’ internal cognitive models, or to models of their teammates’ perceptions of the agent themself as an artificial teammate (e.g., third-order ToM attributions). These capabilities are mostly subconscious in humans, but are especially evident in high-performing teams, where members of the team need to develop internal models of their teammates (e.g., shared mental models or transactive memory systems^5^). In most teams, human members differ in a variety of ways, ranging from the attitudinal (e.g., collective orientation), to the cognitive (e.g., knowledge and expertise), with their internal models containing various aspects of a given teammate such as form of existing knowledge and level of expertise, assigned roles and responsibilities, capabilities, current workload, personality, and more. These internal models form the basis for ToM, and the kinds of mental state attributions that allow the team to coordinate in complex and collaborative activities, where they demonstrate the ability to anticipate other team member’s needs, such as timing information pushes and providing backup behavior^16^. It is currently not known if it is feasible, or useful, for agents to acquire this information through multiple interactions in order to learn about the humans with whom they are interacting. Rather, providing an ASI with the relevant information about their human counterparts might help the agent use its AToM to adapt explanations and suggestions to users^17^, and to inform the agent’s AToM. The ASI should be provided with socially, and situationally, useful data that enables them to develop the AToM models necessary for collaboration.

### Profiling to support artificial theory of mind development

Development of artificial theory of mind will require AI to be provided with not just *enough* data, but, more importantly, *contextually relevant* information about their teammates to begin modeling and making attributions about their beliefs and intentions, and predict future actions. We contend that building profiles, an abstract, quantifiable, and machine-readable description of an individual's characteristics, can be used to provide the a priori information an ASI can leverage to be able to inform their internal models of humans. Drawing on concepts and methods from social, cognitive, and organizational sciences, profiles can be developed to help an agent understand human behavior^18^. Building profile models is largely specific to the context for the use of the profile, as it would not be feasible to capture everything about an individual, so models must be developed with the intended purpose in mind. Profiling individuals originated with user research in marketing, but the approach is also widely applied in video game-based research^19,20^, where profiles of players are able to describe and predict various outcomes and performance measures, including at the team level^21^. In addition to profiling related to task performance, previous work has attempted to understand the features of an individual and a team that are most closely related to their success^22^. Relevant profile features necessarily shift as a function of a team’s given task and context, but there is evidence that underlying features of individual’s (and by extension the teams that they form) knowledge, skills, and attitudes may play more reliable roles in determining teamwork behaviors and success. This includes features such as personality^23^ and social skills^22^, which are particularly important in team settings^24^.

The research reported here was conducted to examine factors influencing the successful performance of ad-hoc teams being advised by an agent architecture developed to be socially intelligent. It was part of a large research program, Artificial Social Intelligence for Successful Teams (ASIST), designed as an interdisciplinary initiative bringing together teams of researchers from the computational, social, and organizational sciences to collaborate on the development of AI capable of being a member of a team^25,26^. A foundational feature of the program was that the AI not be designed to support what we described as taskwork (e.g., engage in active/mechanical working tasks that contribute to the completion of team objectives), but, rather, target AI capable of teamwork, and supporting team processes. The ASI was to monitor and provide teamwork relevant advisement as humans engaged in the task. The task was a simulated urban search and rescue (USAR) operations, a complex task designed to require coordination between teammates assigned varying roles and provided with varying resources. The primary focus of our work was to study the impact of pairing human teams with one of several artificial, socially intelligent agents and how profiling human teams affected this.

We present findings detailing our novel approach to profiling ad-hoc teams that captures features of team composition that may influence team process, success, and perceptions of artificial socially intelligent advisors. The profiles gauged team members and teams in their potential for teamwork and their potential to successfully execute the USAR task (see^27^ for a detailed description of the profile components, an overview will follow in the Methods section of this paper). Our primary hypotheses regarding player profiles were that increased individual taskwork potential would be associated with improved performance of the taskwork allocated to a given player’s role and that increased individual teamwork potential would be associated with improved performance of tasks that required communication, coordination, and joint action with team members. Similarly, we anticipated that profiles at the team level, which were the aggregated team level taskwork and teamwork potential, would interact to predict a team’s ability to perform well on taskwork-teamwork measures. The profiles developed in this work were implemented into the cognitive models of the ASI agents (to varying degrees across the agents); however, a specific, detailed description of the agents’ cognitive architectures and how the profiles were implemented across the agents is out of scope for this manuscript. Rather, we discuss the profiles, including potential behavioral markers and characteristics we expected of different profiles, with consideration for the taskwork and teamwork sides of the profiles, provided to the teams of ASI developers, with a focus on their utility as predictors of differences in experimental outcomes and task performance to aid ASI agents in their role as an advisor.

## Methods

The experiment reported here was pre-registered on the Open Science Framework^28^ and the data collected as part of this study and that is used in the analyses presented in this work, has been made publicly available (data available here:^29^). The experiment manipulated the presence of an ASI teammate serving in an advisory role such that some USAR teams had no advisor, others a human advisor, and the remainder paired with one agent imbued with artificial social intelligences (ASI). The purpose of this at the ASIST programmatic level was to test the effectiveness of six different ASI agents, developed by independent research teams, and as part of a research program testing the effectiveness of social-cognitive architectures developed for teams^25^. Because our interests were more in understanding how profiles are related to human-agent teaming, for our analyses, we collapsed across the six agents combining them into a group of ASI advised teams. We will discuss agent capabilities and constraints in a later section, but overall, the ASI advisors were designed to closely monitor the actions and communications of the teams with which they operated. Based upon inferences derived from the agents AToM, developed by monitoring their human teammates, the agents provided advice and interventions to improve the team’s process and achieve successful outcomes.

### Simulated USAR missions

Teams had to complete two simulated Urban Search and Rescue (USAR) missions executed in a testbed built using the Minecraft game environment (see^29^ for a full description of the testbed). The testbed was designed to support virtual collaboration, allowing for remote experimentation during the pandemic. As such, teams were not collocated, and experiments were coordinated by remotely connecting participants to the testbed along with virtual meeting software allowing for synchronous communication and video of each team member. Missions were completed in a fixed order with each mission featuring a rescue in a partially collapsed building and included a different perturbation such that, in the first mission, teams experienced new rubble falling into the search area, blocking some areas, and, in the second mission, teams experienced a disruption (or black-out) of a shared map included to support coordination. Each of 113 teams were assigned to one of eight (between-team) conditions, with 15 teams in the No Advisor condition, 14 teams in the Human Advisor condition, and 14 teams in each of the remaining six Artificial Social Intelligence Agents (ASIs) Advisor conditions (i.e., 84 total teams in the ASI advisor condition). Each of the three team members were randomly assigned a role, such that the role assignment order for each team was fixed and distributed in order of connecting to the testbed). The three roles in this study were Medic, Engineer, and Transporter, and each role had unique capabilities, tools, and knowledge related to possible locations of victims. Teammates communicated with each other using virtual meeting voice communications as well as through what we call knowledge externalization tools. These tools were designed to be scaffolding for coordinative communications; that is, testbed tools that afforded externalization of cognition^30^ via physical markup inside the virtual building and reflected on a mini-map layout of the USAR environment^31^ (e.g., blocks marked with pertinent information and placed in front of a room in the building).

#### Medic role

One individual on each team was assigned the role of Medic, which involved using a device to triage the building-collapse victims that were distributed throughout the environment to determine what kind of injuries they had. The medic was the only role that could acquire information about whether a victim was type A—abrasions, B—bone damage, or C—critical. After the injuries of a given victim were identified, the medic could then stabilize that victim in preparation for transport. It is relevant to note that victims could be transported before they were stabilized, and that transporting victims was a capacity of all roles on the team; however, the nature of a given victim’s injuries dictated their evacuation point, so the effectiveness with which the Medic acquired and shared this information was foundational to team success as well as efficiency of coordination. Additionally, critical victims required assistance from team members to heal/stabilize, which will be discussed in more detail at the end of this section.

#### Engineer role

A separate individual on each team was assigned the role of Engineer. Engineers had the slowest base movement speed on the team and were able to break rubble blocks. Clearing rubble was a critical task for mission success because it could open new paths or reveal that a victim was trapped within a pile of rubble. Engineers were also uniquely provided with information about the structural stability of rooms in the USAR environment, and they are shown the locations of ‘threat rooms’ (rooms where rubble was likely to fall and trap teammates) on the shared map. The effectiveness with which they shared this information was important for supporting the risk management process of the team.

#### Transporter role

The final individual on each of the three-person teams was assigned to the Transporter role which had the fastest base movement speed on the team, and provided participants with the ability to detect at a distance whether there was a victim inside of a room in the environment. The Transporter’s effectiveness at searching the environment for victims and communicating those locations to their team was vital to the team’s ability to coordinate triage, stabilizing, and evacuation work as well as to organize the interdependent team tasks.

As noted above, all of the roles were able to pick up the victims, carry them and set the victims down in the environment, but the victims would only count as “evacuated” for the team if the victim was both successfully stabilize and transported to the correct evacuation zone for that victim type (A, B, or C). Additionally, each participant was provided with the same set of knowledge externalization and communication tools that displayed various symbols and could be placed on the virtual floor (as well as removed) such that teammates could see them in the environment as well as view them on a shared mini-map. The semantic meaning of each marker block was as follows: Victim type A, Victim B, No Victim Here, Critical Victim, Regular Victim, Threat Room, Rubble, and Help Me Here. These allowed teammates to quickly and clearly communicate their task needs and organize interdependent tasking as well as backup behaviors.

Teams were also challenged with a shared, interdependent joint-task that required two teammates to work together to stabilize critical victims. Although the medic role was still required to stabilize a critical victim, an additional teammate was required to be in proximity to the victim in order for that stabilization to be successful. Walking away from the victim or not being close enough would cause the medic’s stabilization action to fail.

Before completing the experimental trials, participants responded to surveys and measures that captured participant individual differences and current dispositions, and after the missions they completed measures regarding their team’s success, perceptions of team process, and ratings of their team’s advisor (in conditions that included an advisor). The surveys relevant to this manuscript are described in more detail below.

### Materials and measures

#### Individual player profiles

Player Profiles are based on a six-component model that is constructed from psychometric, psychographic, and skill elicitation measures that tap individuals’ taskwork-related and teamwork-related potential. Related to what was described earlier, this distinction is based on team theory to differentiate the varied competencies associated with completing a task and those needed to collaborate effectively. The combined model of the player profile includes six components with three tapping taskwork potential and three tapping teamwork potential. This integrated approach attempts to bridge the gap between traditional approaches to understanding/facilitating human behavior and modern methods for implementing artificial agents.

In this study, taskwork potential refers to a set of largely task generic competencies related to performing in a virtual world. They capture one’s ability to navigate and recall pathing, comfort/familiarity with task completion in game-based environments, and task execution in the custom Minecraft testbed; the three components used in constructing the task potential part of the model were intended to capture these facets. Ability to navigate and recall pathing was captured using the Santa Barbara Sense of Direction (SSOD), a validated measure of spatial navigation^32^ and predictive of ability to successfully learn and navigate both real and virtual environments^33^. Comfort and familiarity with task completion in game-based environments was captured using a Video Game Experience Measure (VGE; see Appendix B of the Study 3 Preregistration in:^29^) which targeted video gaming specific experience and skills related to Minecraft and the USAR gamified task. The third component of the task potential part of the model was more task specific. It was captured through a timed, in-game Competency Test (Comp). Although somewhat task specific, the behaviors were fairly generic in the Minecraft game environment; that is, this was a behavioral measure where each player had to individually complete a task battery requiring that they execute essential game actions necessary to complete the task in the Minecraft testbed (e.g., breaking walls).

The other half of the player profile model, the teamwork potential profile, consists of a set of team generic competencies related to collaboration and interpersonal competencies. This was built to capture an individual’s ability to discern mental states/emotions, group interaction tendencies, and collective engagement and grouping behavior. Ability to discern mental states/emotions was captured using the Reading the Mind in the Eyes Test (RMET). This is a validated measure designed initially to detect subtle deficits in ToM in adults with high-functioning autism, and has also been related to neurotypicals’ ability to make mental state attributions^34^. Interaction tendencies were captured through the Sociable Dominance scale (SD), a validated measure of sociable dominance in individuals that can predict social interactions; for example, it has been found that individuals high in sociable dominance tend to use reasoning and direct communication strategies with others^35^. Finally, collective engagement and grouping behaviors were captured using the Psychological Collectivism scale (Collectivism), a validated measure of attitudes individuals have about working in groups, including preferences for being in a group, concern for the group, and whether they tend to comply with group norms and rules^36^.

**Figure 1:**
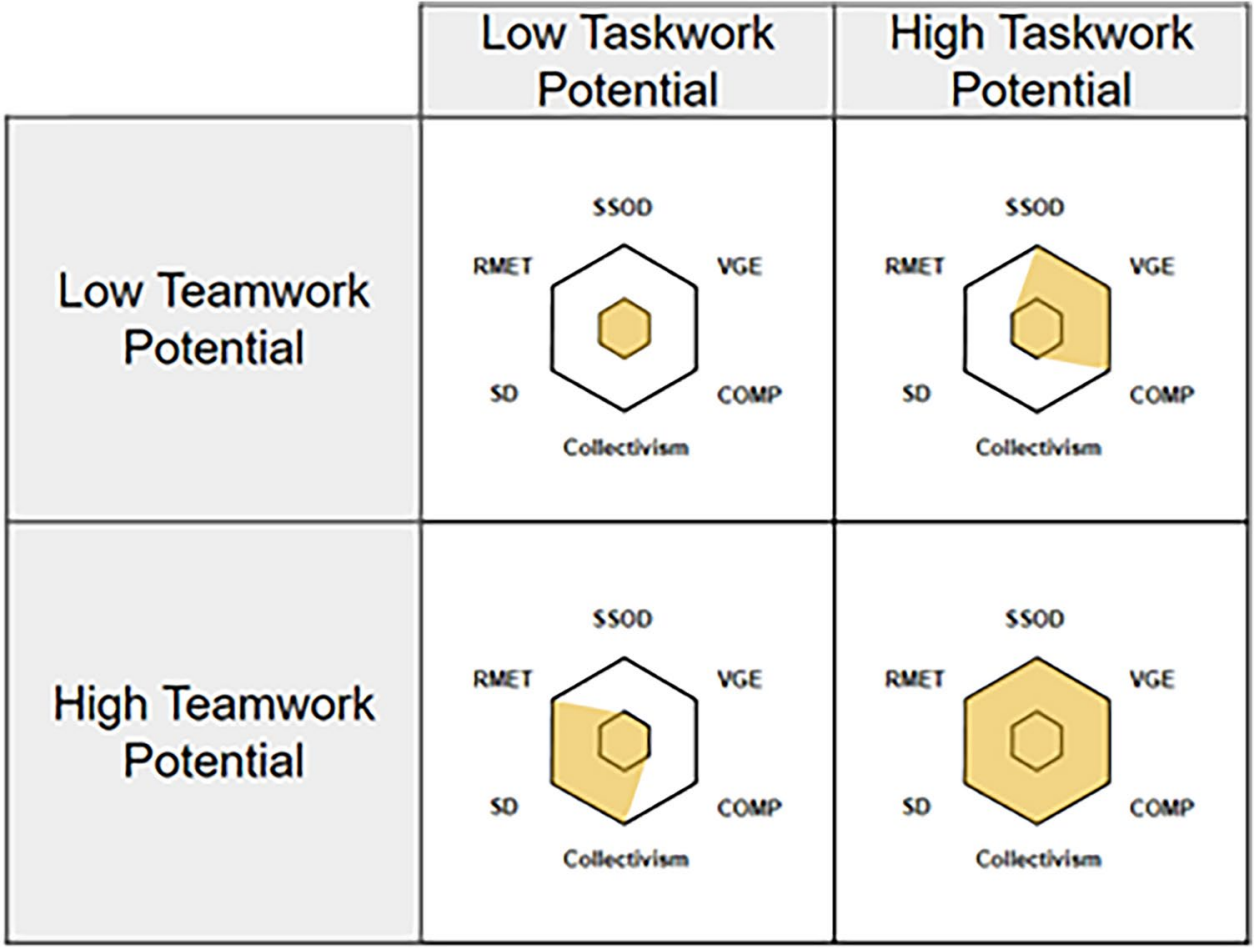
The two dimensions of our profiling approach were Taskwork Potential and Teamwork Potential each defined by scores on three measures. Taskwork potential was determined by: spatial ability (SSOD: Santa Barbara Sense of Direction), video gaming experience (VGE: Video Gaming Experience), and a task skills competency test (COMP: Competency test). Teamwork potential was determined by: preferences for team and group work (Collectivism: Psychological Collectivism), attitudes towards social interactions (SD: Sociable Dominance), and social intelligence (RMET: Reading the Mind in the Eyes Test). Team members were categorized as either high or low in potential on each dimension. The four cells in this graph demonstrate the most extreme possible categorizations with team members in the upper left quadrant demonstrating low potential on both dimensions, and members in the lower right demonstrating high potential on both.

**Figure 2:**
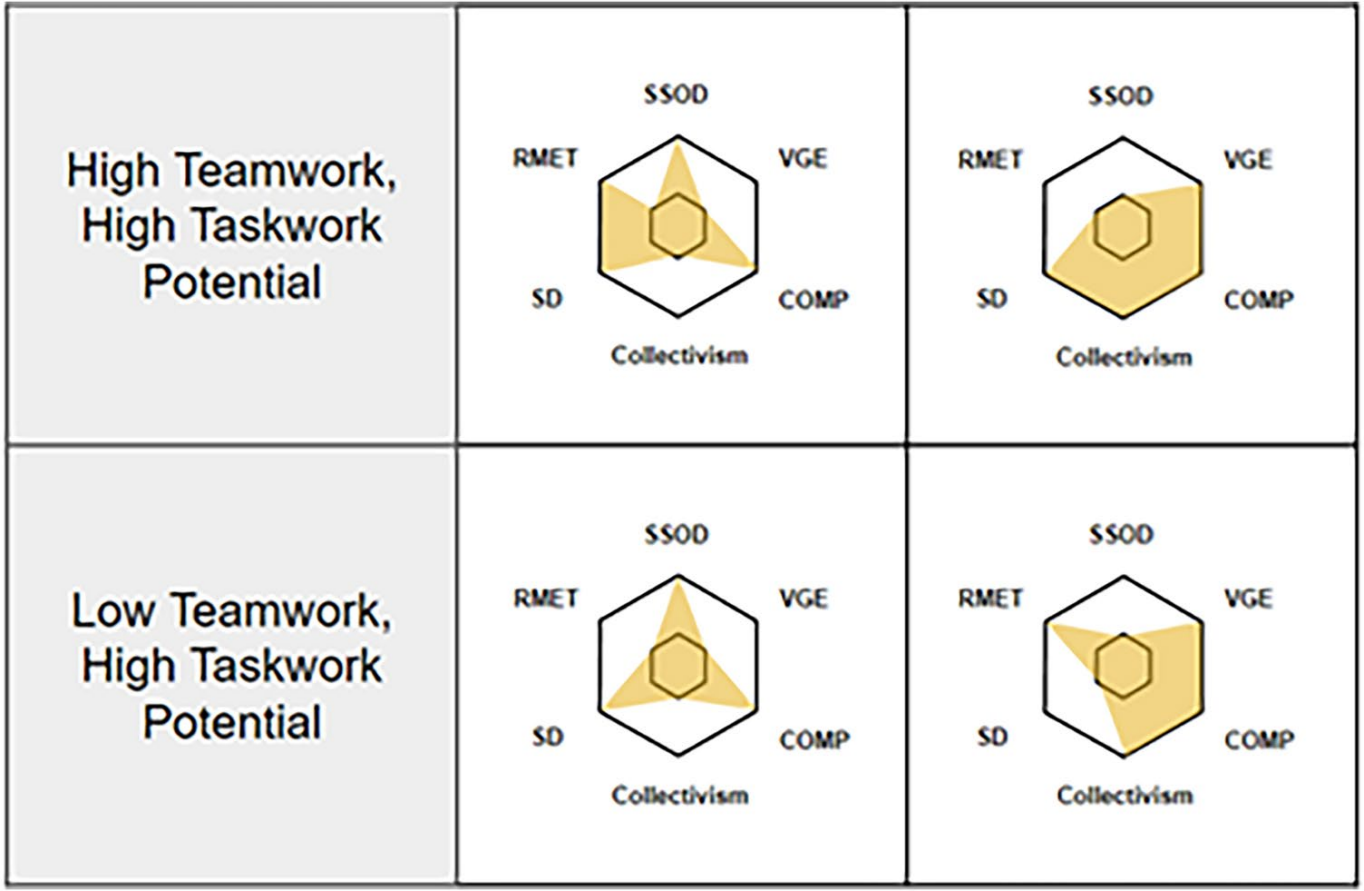
Team members were categorized on Taskwork Potential and Teamwork potential based on their scores relative to the median of the population scores on each measure comprising each dimension. To be categorized as high potential in with Taskwork or Teamwork, participants had to score above the median on at least two of the three measures for the respective dimension. The top two radar charts demonstrate different distributions of high/low scores a participant might produce while still being categorized as high on both dimensions. The bottom two radar charts demonstrate different scores that could possibly result in being categorized as low in Teamwork Potential, but high in Taskwork potential. Note that this is not an exhaustive listing, but rather examples of possible scoring combinations.

Individuals were categorized as high or low in teamwork potential and in taskwork potential based on the combination of their scores on the measures related to that part of the model (see Fig. 1). Specifically, if an individual scored higher on two out of the three measures they would be classified as high, and if they scored low on two out of the three measures they would be classified as low. Possible profile groups included: low taskwork - low teamwork, high taskwork - low teamwork, low taskwork - high teamwork, and high taskwork - high teamwork potential. To provide a concrete example, an individual who scored above the sample median on Video Game Experience measure and above median on the SSOD, would be classified as ‘high’ on task potential. And if that individual scored lower than the median on Sociable Dominance and lower on Reading the Mind in the Eyes, they would be classified as “low” on team potential (see Fig. 2 for examples). Thus, their combined profile would be high taskwork - low teamwork potential.

#### Team holistic profile formulation

Team profiles were constructed from the combination of individual team member profiles through modal analysis (e.g., the most prevalent taskwork and teamwork potential profiles determined the overall team profile). This allowed us to categorize each entire team as low or high taskwork potential and low or high on teamwork potential in a similar manner to the player classifications. For example, if a team consisted of one player that was categorized as high taskwork but low teamwork, a second that was low taskwork low teamwork, and a third that was classified as high taskwork high teamwork, they would then be collectively categorized as high taskwork (2 of 3 representatives) low teamwork (2 of 3 representatives). Further, to provide more insight into the predictiveness of the profiling technique we additionally provide analyses focused on the far ends of the classification spectrum (e.g., teams that were classified as low taskwork - low teamwork or as high taskwork - high teamwork). These groups were selected for additional analysis because this approach to holistically profiling teams is novel, and we hypothesize that there may be interactions between the teamwork and taskwork profiles that would make interpretation of the low taskwork - high teamwork, high taskwork - low teamwork teams unclear at best.

#### Artificial social intelligence agents overview

The six ASI agents in this study were individually developed by ASIST program performers/teams, and were instantiated with a performer-defined AToM, implemented as computational models of team attributes, teamwork processes, and their impact on effects in teams (see^29^ for these descriptions in the study preregistration). The ASI agents acted in an advisory role to support their three human team members with teaming behaviors and engaging in teamwork in the experimental tasks. The agents were required to adhere to certain constraints to maintain the primary goal of supporting teamwork processes rather than taskwork – to this end, the agents were not embodied in the virtual Minecraft-based simulated USAR testbed and were not able, or allowed, to engage in any of the taskwork in this study. The ASI were also constrained with respect to their knowledge of the environment so that it would comparably be realistic in the real world. Specifically, they were not given omniscient knowledge of where every victim was, what the best route to a particular location would be, or where threats (e.g., risk of further building collapse) may exist. However, the ASI were able to observe the actions the human team members took in the virtual environment, see the externalized cognitive artifacts (e.g., marker blocks), and see the field of view for each team member to allow them to perceive what each person has seen or encountered in the environment. This allowed the ASI agents to make inferences about the human team members’ beliefs, intentions, goals, and knowledge based on the observable actions taken in the environment. The different agents used various approaches to this, some utilizing Bayesian approaches to model human perspectives, short-term and long-term planning, aspects of human cognition such as workload or emotion, and track multiple hypotheses^37^. Other agents use internal models that estimate participant knowledge of relevant known entities spatially through a 2D representation of the experimental environment, using neural network prediction models developed on human-annotated and simulated player data to make inferences and predictions based on accumulating knowledge of, and modeling, each individual team member over time and the team as a whole^38^. Additionally, the agents were provided access to all surveys taken by participants before completing the experimental task, as well as various analytic components (ACs) that were developed independently by performer teams. To varying degrees, the ACs were developed based upon team theory and designed to augment the ASI architecture. As described, our AC was developed to help the ASI understand player profiles. Other research teams based their ACs on, for example, leadership theory. For example, one AC used pre-experiment surveys and in-game data to identify emergent leadership in human players^39^. This could then be used by ASI to determine where to direct leader relevant interventions.

The player profiles we described above were implemented in the testbed as an analytic component with the intent to provide machine-readable input about the players, and the team as a whole, through the quantified profile model. The player profile analytic component read the survey and gameplay data used in the model, calculated the profile for each individual and the team overall, and published the player profile model’s output to the message bus used by the agent to receive testbed, survey, and component data. The player profile models were developed during prior program studies (see^25,40^) before the inclusion of the ASI agents in the present study. The profiles afforded the ASI the ability to interpret the measures and gameplay data used in the profile construction in the context of the theoretical grounding used to develop the profiles over the course of the ASIST program. The ASI agents integrated the player profile data into their internal models to help inform their predictions, allowing the ASI to consider an individual’s potential capacity for teaming and tasking behaviors into their existing models and calculations. Specification of the technical integration of the player profile models, or any analytic component used in this study, into the various ASI agents AToM and cognitive architectures is beyond the scope of this paper. Interested readers are referred to the publicly available dataset, which also contains all of the code and documentation for the agents used in this study, and for the documentation of the player profile analytic component (see^27^). Additionally, this study did not manipulate the provision of the player profiles or any other information to the ASIs as a variable. All ASI agents were provided the same access to the testbed, survey, and analytic component output data. Thus, because we were not able to manipulate the provision of profiles within agents and teams, we are unable to comment on the particular impact the profiles had on agent interventions and determinations provided to teams. Rather, our analyses focus on the player profile models predictive power with regards to the experimental task measures and perceptions of the ASI. This allows us to comment on the utility of the a priori information being provided to actual artificial social intelligence agents through the player profile model, and whether they provided the ASI with data that is indicative of overall and specific task performance.

#### USAR task metrics

Performance on the USAR task was tracked along multiple dimensions. Each of these measures are considered to reflect successful taskwork, successful teamwork, or a combination of taskwork and teamwork.

At the individual level, each role that participants could be assigned was associated with a set of unique or optimal taskwork functions that they could perform as well as a set of teamwork functions in which they could engage to support coordination. Given the nature of the testbed, measures of teamwork were relatively difficult to track because, as mentioned above, human teammates communicated through voice comms, and the natural language associated with those exchanges has not yet been fully processed and analyzed by our team. Accordingly, the measures we employ for this article often are taskwork focused or have taskwork woven into their execution, but several either have teamwork components or are entirely reflective of teamwork actions (see Table 1).

**Table 1:** Outcome measures related to the execution of taskwork and teamwork elements of teams’ simulated missions.

Measure name	Measure description
*Taskwork Performance Measures at the Individual Level*	
*Engineer taskwork measure 1: rubble destroyed*	Due to the straightforward nature of the Engineer role, the primary measure available for evaluation of role performance is simply the number of rubble blocks that an engineer cleared in the environment. This measure is considered to reflect only taskwork because while it is possible that the engineer cleared rubble in response to teammate requests for assistance, it is not possible to distinguish these events with our current data
*Performance Measures on Taskwork-Teamwork at the Individual Level*	
*Medic taskwork-teamwork measure 1: critical victims healed*	Healing critical victims is notably distinct from healing regular victims due to the interdependency requirement involving coordination with another team member. Accordingly, the number of critical victims healed is considered to reflect a combination of taskwork and teamwork effectiveness such that more critical victims healed is associated with better performance
*Transporter taskwork-teamwork measure 1: critical victims moved*	Although all team members could pick up and move victims, the transporter moved at a far faster speed and was therefore optimized for searching the environment and moving victims. Due to the influence of team coordination efforts on transporter behaviors as well as the interdependent actions required for the stabilization of critical victims, this measure is considered to represent a combination of taskwork and teamwork
*Transporter taskwork-teamwork measure 2: critical victims evacuated*	There is an important distinction between the movement of and evacuation of critical victims. To be evacuated by a transporter, a critical victim must first be stabilized by the medic (in tandem with an additional teammate, possibly the transporter) and then moved to the correct evacuation point by the Transporter. Therefore, the movement of a critical victim represents a first stage coordinated team action whereas evacuating a critical victim represents a third stage coordinated action and therefore a combination of taskwork and teamwork
*Perception Measures at the Individual Level*	
*Individual team perception measure 1: team process rating*	At the end of each mission, participants were given the opportunity to respond to a scale on which they rated their perceptions of elements of their team’s process (Mathieu et al., 2020). Each individual’s averaged response is employed here as a measure of their team perceptions
*Individual ASI advisor perception measure 1: advisor was dependable*	At the end of each mission, participants were also asked to rate their advisors (if they had one) along a set of dimensions including the degree to which they felt their advisor was dependable. Responses to that query are used in this report as a measure of individual’s perceptions of the ASI advisor in particular
*Individual ASI advisor perception measure 2: advisor was reasonable*	An additional query regarding ASI advisors tapped whether participants thought that the advice provided by the ASI was reasonable or based on reason. We employ responses to that query here to capture individual’s perceptions of those ASI advisors
*Performance Measures on Teamwork at the Team Level*	
*Team teamwork measure 1: cost of risk management errors*	As described above, participants could become trapped in rooms that were known to be prone to structural collapse. When trapped, participants could only be extracted by the assistance of the engineer role and were otherwise unable to continue through the environment to execute their mission. The time lost to these entrapment events is calculated here as the cost of risk management errors in milliseconds and considered to reflect primarily team’s teamwork effectiveness due to the need to communicate the location of threat rooms as well as to manage the risk that they pose
*Team teamwork measure 2: use of knowledge externalization tools*	All participants on a team had the ability to utilize the same set of knowledge externalization tools to communicate the knowledge they gathered in the environments, their tasking and coordination needs, and to broadcast requests for assistance. The usage of these tools involved both the placement and removal of markers as it was found to be just as important to remove outdated markers as to place pertinent markers. The overall employment of these tools was considered to be an indicator of a team’s teamwork effectiveness
*Performance Measures on Taskwork-Teamwork at the Team Level*	
*Team taskwork-teamwork measure 1: mission score*	Overall team performance was calculated as a function of the number and type of victims that were successfully stabilized and transported to their appropriate evacuation point; we present that measure here as a percentage of possible mission score. Mission score is considered to reflect a combination of taskwork and teamwork at team level and will be reported in this manuscript as a combined taskwork-teamwork indicator
*Team taskwork-teamwork measure 2: heal-to-extract rate*	The overarching flow of the team’s task required searching for, triaging, stabilizing, and evacuating victims. Whereas overall mission score reflects the final successful outcome of evacuation, this heal-to-extract rate reflects the number of times that a victim was stabilized, but not successfully extracted. We consider this to be a combined measure of a team’s taskwork and teamwork effectiveness
*Perception Measures at the Team Level*	
*Team-level team perception measure 1: team process rating*	The team-level team process rating represents the average responses of the individuals on a team referring to their perceptions of teamwork quality
*Team-level ASI advisor perception measure 1: advisor was dependable*	The team-level ASI dependability rating represents the average responses of the individuals on a team on how dependable they perceived the ASI to be
*Team-level ASI advisor perception measure 2: advisor was reasonable*	The team-level ASI reasonable rating represents the average responses of the individuals on a team on how reasonable they perceived the ASI to be

### Study sample

This study involved 113 three-person teams completing two 17-minute gamified urban search and rescue missions implemented in Minecraft. All participants engaged in the study remotely from an internet connected computer of their choice and were overseen by experimenters at Arizona State University (see the Study 3 Preregistration in: ^29^) who carried out the methods in accordance with the approved protocol, and relevant guidelines and regulations. All participants in this study reviewed and completed an informed consent form prior to participation. The study was reviewed and approved by the Arizona State University Institutional Review Board. The data sharing agreement and approval for data analysis by researchers at the University of Central Florida were overseen, reviewed, and approved by the University of Central Florida Institutional Review Board. All local approvals were further submitted to the Army Human Research Protections Office (AHRPO) for supplemental review.

#### Analysis populations

The analyses reported here relate to multiple different groups of participants as a function of the focus on individual versus team outcomes, the availability of complete data for player profiling, the availability of complete data for team profiling, and the exclusion of groups to support the inspection of outcomes related to ASI advisors specifically. More detailed study information on this study and the data repository can be found at^28^ and^29^ respectively. See Table 2 below for demographics descriptives for each subset of the data.

**Table 2:** Descriptive features of each dataset analyzed in the following results section.

	Profiled Participants	Participants on Profiled Teams	Participants on Advised, Profiled Teams (Human or ASI; 84 teams)	Participants on Teams Advised by ASI (70 teams)	Participants on Teams Profiled as Low-Low or High-High (53 teams)
Average age (SD)	22.86 (6.09)	22.82 (5.84)	23.15 (6.14)	23.46 (6.02)	22.79 (6.67)
Male	254	216	185	154	118
Female	75	69	59	48	41
Total	335	291	249	207	162

## Results

We report the results of our analyses in two parts: first, we examined the outcomes related to individual players’ profiles and their respective performance as teammates, and second, we examined the team-level outcomes with particular attention to the impacts of teaming with an autonomous, socially intelligent agent. At the individual player level, we tested sets of hypotheses related to the interaction of the teamwork and taskwork components of our player profiling approach. Those interactions were also tested at the team level, but, in addition, we tested the interaction of teams’ holistic profiles with the type of advisor that assisted them in their missions. It is important to note that we only considered players’ and teams’ performances and outcomes associated with the second mission that they completed (see Methods), and that analyses focused on the artificial socially intelligent agents necessarily incorporated only those teams which interacted with them. Full details of the analyses reported here including descriptive statistics are available in the supplementary information (see file: Supplementary Information.docx, also available at: ^25^).

Shapiro-Wilke Tests were run for the dependent variables, and though the results were significant for a subset, inspection of the Kurtosis and Skewness ratios indicated that these deviations were small (e.g., no more than a ±4 Skewness or Kurtosis ratio, which is greater in magnitude than the ±2 standard, but is not extreme deviation) and we proceeded with analysis of variance as our sample sizes are generally considered sufficient to ensure that ANOVAs are likely to be robust to those assumption violations. All of the following analyses employ an alpha cutoff of *p* < 0.05, and interpretations of effect size are based on field standards such that *η*^2^ < 0.06 is considered a small effect, between 0.06 and 0.14 and is considered a moderate effect, and *η*^2^ > is considered a large effect^41^.

### Individual player profiles

Our primary hypotheses regarding the player profiles were that increased individual taskwork potential would be associated with improved performance of the taskwork allocated to a given player’s role and that increased individual teamwork potential would be associated with improved performance of tasks that required communication, coordination, and joint action with team members (see Table 3). Additionally, we hypothesized that players demonstrating increased teamwork potential would report more positive perceptions of their teams and their advisors (see Table 4). Unless otherwise stated, the analyses reported here used α = 0.05 cut-off. To test performance on individual’s taskwork across the individual teamwork and taskwork potential profiles, a two-way ANOVA was conducted with each of the role-respective measures.

**Table 3:** Individual profiles: teamwork measure analyses. These results demonstrate that the taskwork and teamwork dimensions of the profiles separately predict differences in individual task and team activity performance.

Role	Metric	Taskwork profile	Teamwork profile	Taskwork × Teamwork interaction
Medic	Medic taskwork-teamwork measure 1 (critical victims healed)	F(1, 105) = 7.81 *p* = 0.006 *η*^2^ = 0.067^++^	F(1, 105) = 0.44 *p* = 0.509 *η*^2^ = 0.004	F(1, 105) = 1.20 *p* = 0.276 *η*^2^ = 0.010
Engineer	Engineer taskwork measure 1 (rubble destroyed)	F(1, 103) = 0.64 *p* = 0.425 *η*^2^ = 0.006	F(1, 103) = 0.06 *p* = 0.804 *η*^2^ = 0.0006	F(1, 103) = 1.27 *p* = 0.262 *η*^2^ = 0.012
Transporter	Transporter taskwork-teamwork measure 1 (critical victims moved)	F(1, 105) = 0.58 *p* = 0.448 *η*^2^ = 0.005	F(1, 105) = 5.60 *p* = 0.020 *η*^2^ = 0.050^+^	F(1, 105) = 0.58 *p* = 0.448 *η*^2^ = 0.005
Transporter	Transporter taskwork-teamwork measure 2 (critical victims evacuated)	F(1, 105) = 0.299 *p* = 0.586 *η*^2^ = 0.003	F(1, 105) = 4.06 *p* = 0.046 *η*^2^ = 0.037^+^	F(1, 105) = 0.026 *p* = 0.873 *η*^2^ = 0.0002

^+^small effect, ^++^moderate effect, ^+++^large effect.

**Table 4:** Perceptions of advisors as a function of player profile dimensions. Note that row 1 reflects all players that received advisement (either from human or ASI advisors), and row 2 and 3 reflect players that worked specifically with an ASI. These findings highlight that the profile dimensions (taskwork and teamwork potential) predict differences in players’ overall team process perceptions, and that the two dimensions interact to predict differences in perceptions of ASI advisors.

Role	Metric	Taskwork profile	Teamwork profile	Taskwork × Teamwork interaction
All roles, all advised teams	Perceived Team Process Quality	F(1, 274) = 5.980 *p* = 0.015 η^2^ = 0.021^+^	F(1, 274) = 5.851 *p* = 0.016 η^2^ = 0.020^+^	F(1, 274) = 0.172 *p* = 0.679 η^2^ = 0.0005
All roles, ASI advised teams	Advisor dependability	F(1, 211) = 0.055 *p* = 0.815 *η*^2^ = 0.0002	F(1, 211) = 1.59 *p* = 0.208 *η*^2^ = 0.007	F(1, 211) = 1.138 *p* = 0.287 *η*^2^ = 0.005
All roles, ASI advised teams	Advisor reasonable	F(1, 211) = 0.121 *p* = 0.729 *η*^2^ = 0.0005	F(1, 211) = 0.884 *p* = 0.348 *η*^2^ = 0.004	F(1, 211) = 8.447 *p* = 0.004 *η*^2^ = 0.038

^+^small effect, ^++^moderate effect, ^+++^large effect.

#### Individual player profiles and performance

For measures of individual taskwork-teamwork performance (e.g., Medic taskwork-teamwork measure 1, Transporter taskwork-teamwork measure 1) the effect of individual *teamwork* potential profile was found to be statistically significant, indicating that participants with high teamwork potential profiles performed better on taskwork-teamwork measures as compared to those with low teamwork potential profiles (see Table 3). Additionally, for one of these measures (Medic measure 1, critical victims healed) individual *taskwork* potential profile was found to be significant with a moderate effect size such that participants with high taskwork potential outperformed those with low taskwork potential. In contrast, *taskwork* potential profile was not found to be predictive of the taskwork measure for one of the roles (Engineer taskwork measure 1). Last, there was no significant interaction effect between individual *taskwork* potential profile and individual *teamwork* potential profile on the individual performance measures which indicate that *teamwork* potential and *taskwork* potential may have influenced wholly separate components of teammates’ tasking and coordinative capacity.

#### Individual player profiles and perceptions

Outcomes of ANOVAs conducted on individual participants’ perceptions of their team processes and, separately, their ratings of their ASI advisors showed an interesting interplay between the teamwork and taskwork profile dimensions. To be clear, the analysis of team processes incorporates all teams that were profiled whereas the reflections on ASI teammates includes only teams that worked with an artificial socially intelligent advisor. For perceptions of team process, results showed that both *taskwork* and *teamwork* potential profiles predicted team process ratings; however, there was not an interaction between the two, indicating that individuals who were higher in *taskwork* potential viewed their team’s processes more positively and those higher in *teamwork* potential viewed their processes more positively but that the two did not combine to yield substantially higher ratings (or lower ratings in the case of low potentials).

**Figure 3:**
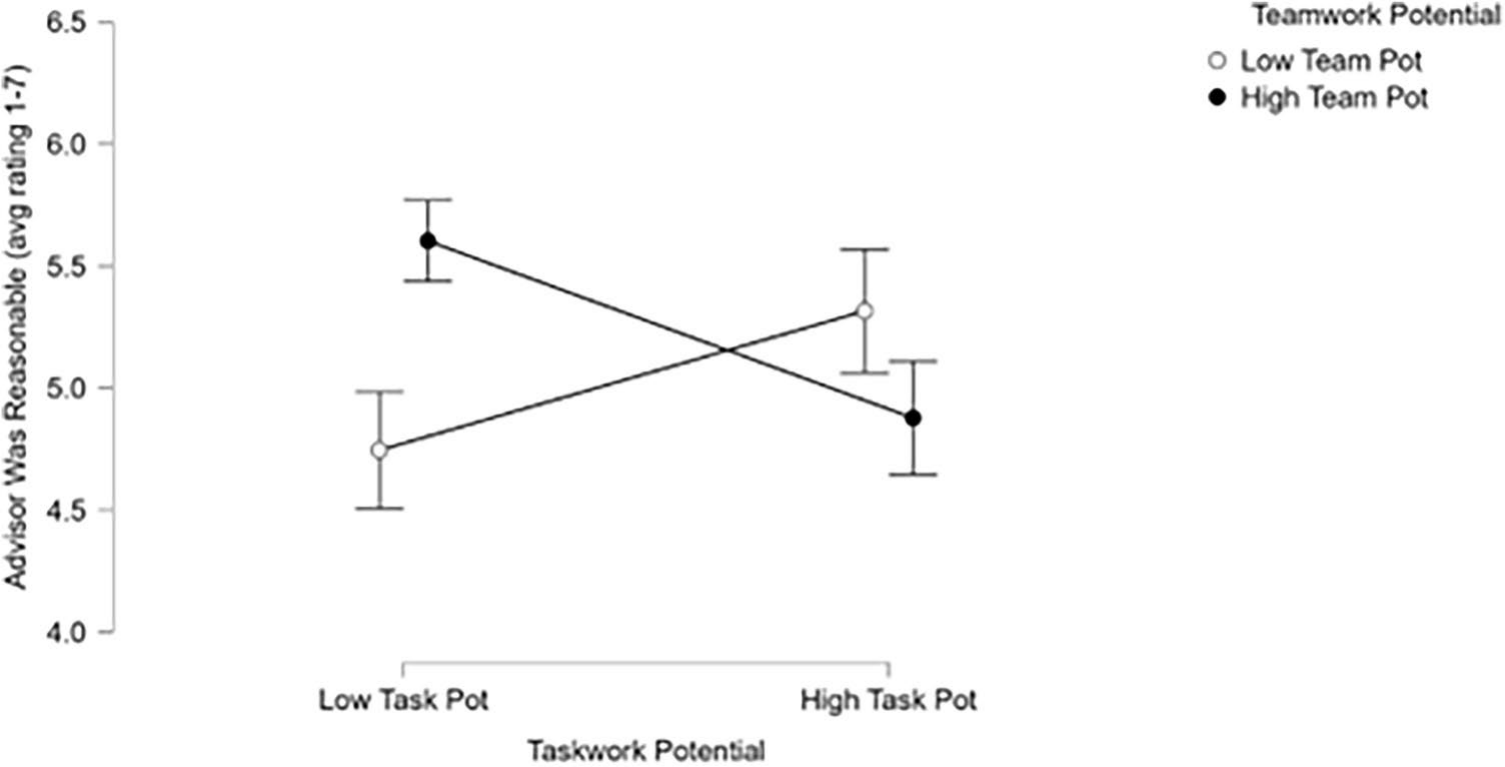
Results showed that there was an interaction between individuals’ teamwork potential profile and taskwork potential profile such that high teamwork but low taskwork potential was associated with the highest ratings of ASI advisors while low teamwork and low taskwork potential was associated with the lowest ratings. Rather, individuals that did not exhibit the skills to succeed on their own but were geared towards teamwork seemed to value the ASI advisors more highly than those teammates that had high taskwork potential.

Participants’ ratings of their advisors were not associated with a particular pattern of results overall. No significant effects were found for *taskwork* potential or *teamwork* potential on participants’ ratings of whether they viewed their artificial socially intelligent advisor to be dependable. In contrast, a significant effect was found for the interaction of *taskwork* potential and *teamwork* potential for ratings of whether participants viewed their ASI advisor as reasonable. Notably, participants with a high *teamwork* potential profile but low *taskwork* potential profile rated their advisors most highly, whereas those with a low *teamwork* potential profile and a low *taskwork* potential profile rated their advisor least highly (see Fig. 3). This suggests that having low taskwork potential coupled with high teamwork potential altered how one valued the ASI contributions.

### Team Profiles

Our prior section examined how profiles, at the individual level, were related to performance. In this section, we describe analyses where those profiles are combined to the team level. That is, here we examine the predictive utility of emergent team profiles generated from modal analysis of the profiles of each of the players on a given team (see Methods). First, we tested the effects and interactions between the teamwork and taskwork components of the team profiles (see Table 5), and second, we examined the effect of team profiles specifically when teams worked with an artificial socially intelligent agent (see Table 6), and then as they manifested with each category of advisor (no advisor, human advisor, and artificial socially intelligent advisors; see Table 7).

**Table 5:** Analyses at the team level show that the interaction of the taskwork and teamwork dimensions of the profile are predictive of performance differences across teams. Particularly, those teams higher in taskwork and teamwork potential demonstrated improved performance outcomes.

Metric	Taskwork profile	Teamwork profile	Taskwork × Teamwork interaction
Taskwork-teamwork measure 1 (mission score %)	F(1, 93) = 4.497 *p* = 0.037 *η*^2^ = 0.043^+^	F(1, 93) = 0.397 *p* = 0.530 *η*^2^ = 0.004	F(1, 93) = 6.122 *p* = 0.015 *η*^2^ = 0.058
Taskwork-teamwork measure 2 (heal: extract error)	F(1, 93) = 0.023 *p* = 0.879 *η*^2^ = 0.0002	F(1, 93) = 0.292 *p* = 0.590 *η*^2^ = 0.003	F(1, 93) = 4.913 *p* = 0.029 *η*^2^ = 0.05^+^
Teamwork measure 1 (risk management failure cost)	F(1, 92) = 0.339 *p* = 0.562 *η*^2^ = 0.003	F(1, 92) = 0.022 *p* = 0.881 *η*^2^ = 0.0002	F(1, 92) = 9.105 *p* = 0.003 *η*^2^ = 0.089^++^
Teamwork measure 2 (knowledge externalization)	F(1, 93) = 0.252 *p* = 0.617 *η*^2^ = 0.003	F(1, 93) = 0.793 *p* = 0.375 *η*^2^ = 0.008	F(1, 93) = 2.796 *p* = 0.098 *η*^2^ = 0.029

^+^small effect*,* ^++^moderate effect*,* ^+++^large effect.

**Table 6:** Team taskwork and teamwork potential: team process and ASI perceptions. Similar to the individual level analysis of ASI perceptions, teams that were collectively lower in taskwork potential but high in teamwork potential demonstrated greater ratings of the ASI advisors.

Metric	Taskwork potential	Teamwork potential	Taskwork × Teamwork interaction
Team Process Rating	F(1, 79) = 1.782 *p* = 0.186 *η*^2^ = 0.020	F(1, 79) = 0.098 *p* = 0.755 *η*^2^ = 0.001	F(1, 79) = 5.633 *p* = 0.02 *η*^2^ = 0.064^++^
ASI dependable	F(1, 65) = 0.001 *p* = 0.973 *η*^2^ = 0.00001	F(1, 65) = 0.877 *p* = 0.353 *η*^2^ = 0.013	F(1, 65) = 1.888 *p* = 0.174 *η*^2^ = 0.028
ASI reasonable	F(1, 65) = 0.296 *p* = 0.588 *η*^2^ = 0.004	F(1, 65) = 1.517 *p* = 0.222 *η*^2^ = 0.021	F(1, 65) = 7.079 p = 0.01 η^2^ = 0.097^++^

^+^small effect, ^++^moderate effect, ^+++^large effect.

**Table 7:** Team holistic profiles and advisor types: mission performance measures. These results highlight the critical finding that ASI advisors significantly improved the performance of low taskwork, low teamwork potential teams.

Measure	Team Holistic Profile	Advisor Type	Team Profile × Advisor Type
Taskwork-teamwork measure 1 (mission score %)	F(1, 48) = 5.812 *p* = 0.02 *η*^2^ = 0.093^++^	F(2, 48) = 0.982 *p* = 0.382 *η*^2^ = 0.031	F(2, 48) = 4.295 *p* = 0.019 *η*^2^ = 0.137^++^
Taskwork-teamwork measure 2 (heal: extract error)	F(1, 48) = 0.443 *p* = 0.509 *η*^2^ = 0.009	F(2, 48) = 0.036 *p* = 0.965 *η*^2^ = 0.001	F(2, 48) = 0.198 *p* = 0.821 *η*^2^ = 0.008
Teamwork measure 1 (risk management failure cost)	F(1, 47) = 4.553 *p* = 0.038 *η*^2^ = 0.076^+^	F(2, 47) = 5.932 *p* = 0.005 *η*^2^ = 0.199^+++^	F(2, 47) = 2.017 *p* = 0.144 *η*^2^ = 0.068
Teamwork measure 2 (knowledge externalization)	F(1, 48) = 4.485 *p* = 0.039 *η*^2^ = 0.068^+^	F(2, 48) = 1.899 *p* = 0.161 *η*^2^ = 0.058	F(2, 48) = 8.395 *p* < 0.001 *η*^2^ = 0.256^+++^

^+^small effect*,* ^++^moderate effect*,* ^+++^large effect.

#### Team profiles and performance

**Figure 4:**
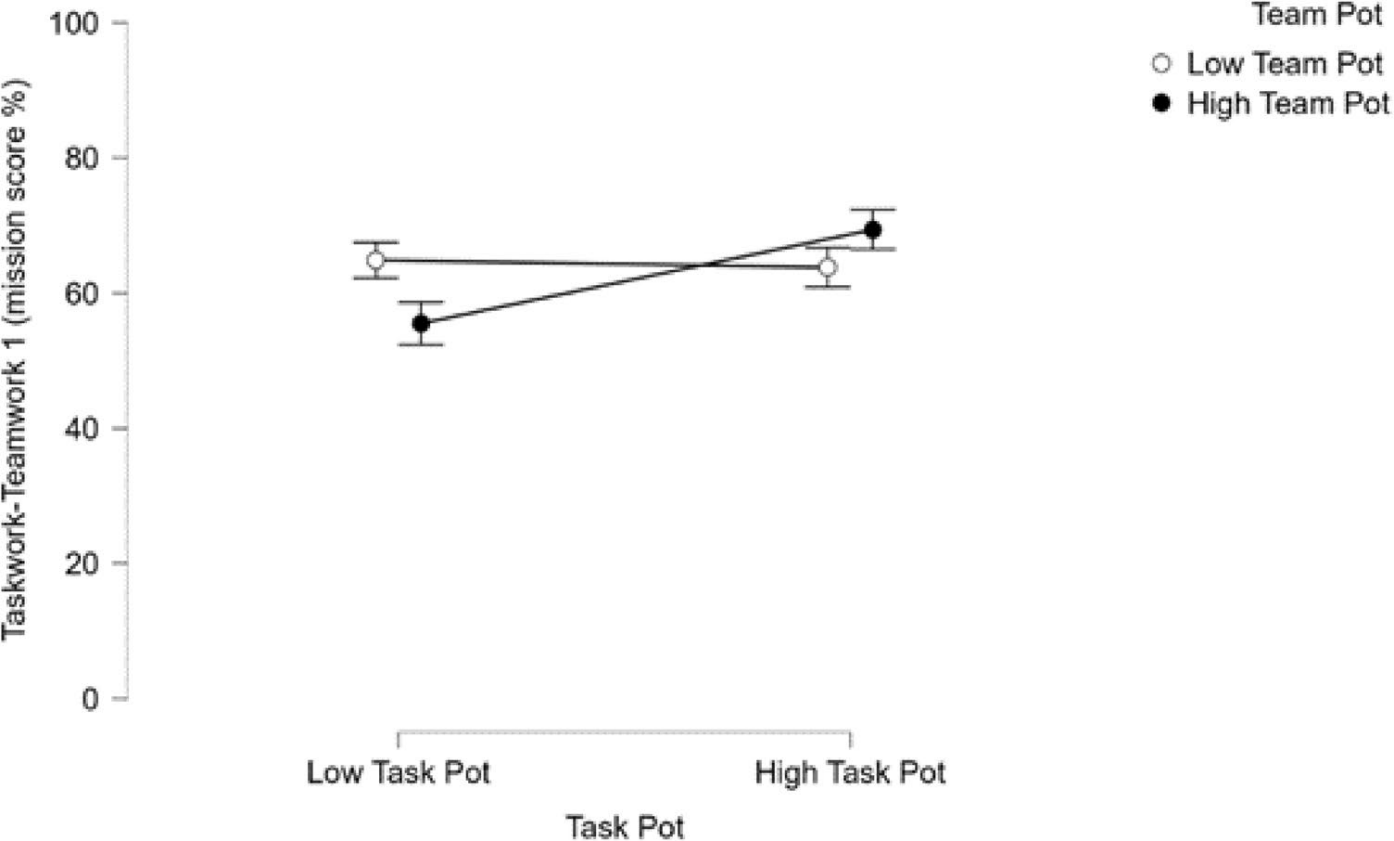
The interaction between teams’ teamwork and taskwork potential profiles was found to account for a moderate proportion of variance in taskwork-teamwork measure 1 (mission score %). Notably, teams that were high in taskwork potential and high in teamwork potential attained higher mission scores, in terms of percent of the maximum possible score.

Outcomes of a series of ANOVAs testing the effects of *teamwork* and *taskwork* potential profiles at the teams level revealed a consistent pattern of results demonstrating that the interaction of *teamwork* and *taskwork* profiles was predictive of team performance outcomes. Although the teamwork-taskwork combined measures employed for evaluating teams’ performances (see Table 5) showed no statistically significant main effect of *taskwork* profile nor of *teamwork* profile, each of the three revealed a significant and small-to-moderately sized effect for the *taskwork teamwork* profile interaction. As anticipated, these analyses revealed that teams that were categorized as high in *taskwork* as well as high in *teamwork* potential performed best overall (see Fig. 4). As predicted, for most measures, teams that were low in *taskwork* and *teamwork* potential performed worst. But, for one measure (mission score %), teams low in *taskwork* potential but high in *teamwork* potential performed worst whereas teams high in both performed best.

#### Team profiles and perceptions

Similar to the pattern of results for team performance, results of ANOVAs conducted to test the effects of team profiles on perceptions of team process and ASI advisors revealed that the interaction of *taskwork* profile and *teamwork* profile predicted differences in participants responses. Again here, the analysis of team processes incorporates all teams that were profiled whereas the reflections on ASI teammates includes only teams that worked with an artificial socially intelligent advisor. For these analyses, there were no main effects of either *taskwork* potential or *teamwork* potential (see Table 6). Notably, although the interaction effect was significant, the pattern of responses across profiles was different than anticipated. Instead of high *taskwork* and high *teamwork* teams responding most positively, the most positive responses were provided by teams that were low in *taskwork* potential but high in *teamwork* potential. This pattern is the opposite of what was found in the analyses of team performance in which low *taskwork* high *teamwork* teams were found to have generally performed worst.

#### Interactions between advisor types and teams’ profiles (low-low, high-high profiled teams)

The prior section demonstrated the utility of our profiling technique when it comes to showing associations between team and task potential and various process and performance measures. We turn next to our examination of the relationship between profiles and process and performance depending on the nature of advising provided the teams. Particularly, we analyze how team process and performance was affected by the presence of either a human or machine advisor imbued with artificial social intelligence. To forecast our findings and provide context to the reader, the primary outcome that these analyses highlight is a positive impact of ASI advisors on low potential teams.

Following the approach described in the most recent section, these analyses tested the effects and interactions between teams’ holistic profiles (e.g., the combination of their taskwork potential and teamwork potential classifications) and the type of advisor with which they worked during their missions (see Table 7). Note that for the following analyses only teams at the extreme ends of the profile dimensions were examined, specifically teams that were categorized as High Taskwork, High Teamwork potential and those categorized as Low Taskwork, Low Teamwork potential.

**Figure 5:**
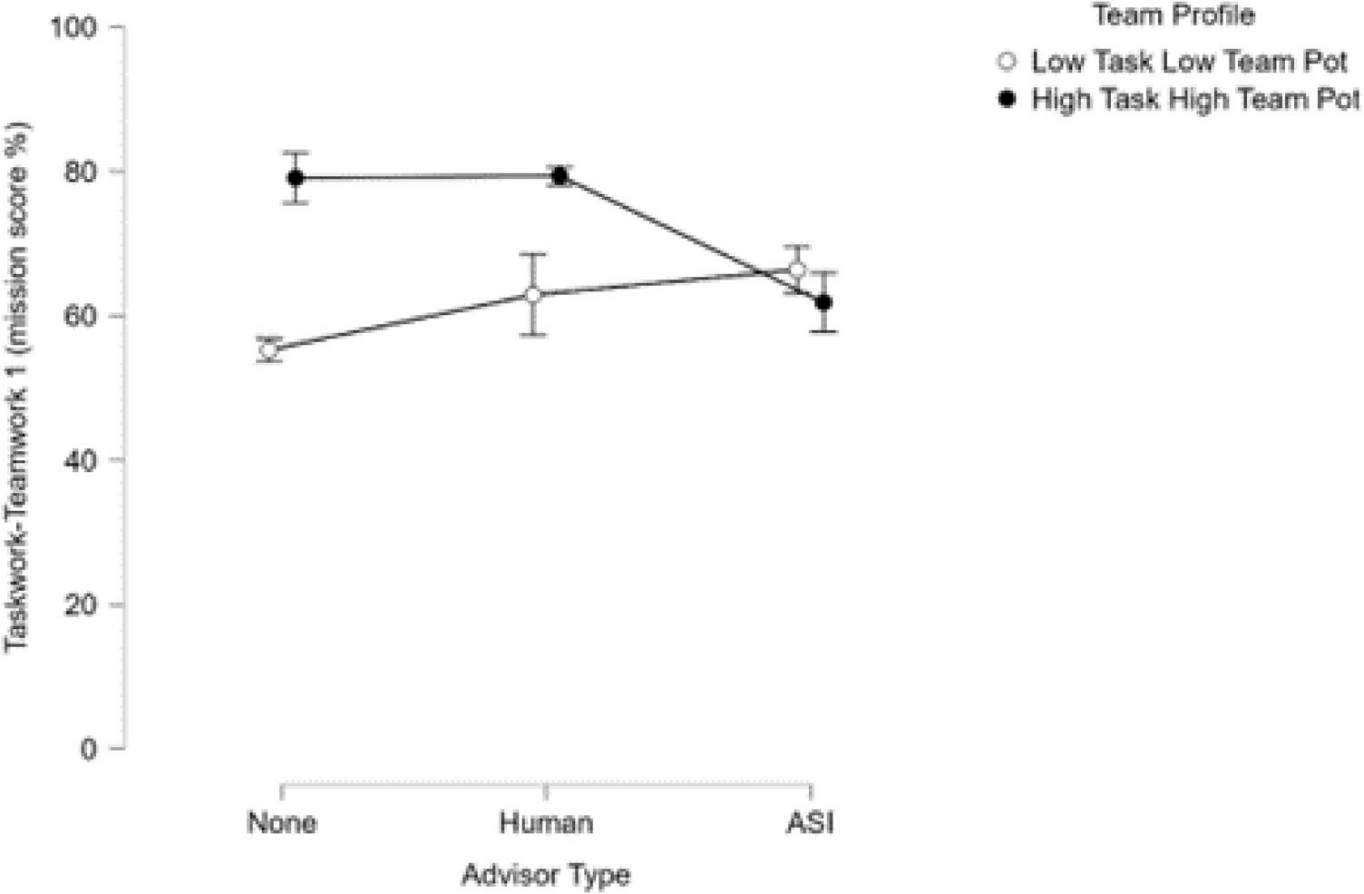
Mission score by advisor condition. Here, the impact of ASI advisors on low taskwork, low teamwork potential teams can be observed clearly. Interestingly, ASI may have also reduced the performance of high taskwork, high teamwork potential teams, highlighting the importance of appropriate assignment and employment of artificially intelligent assistants.

**Figure 6:**
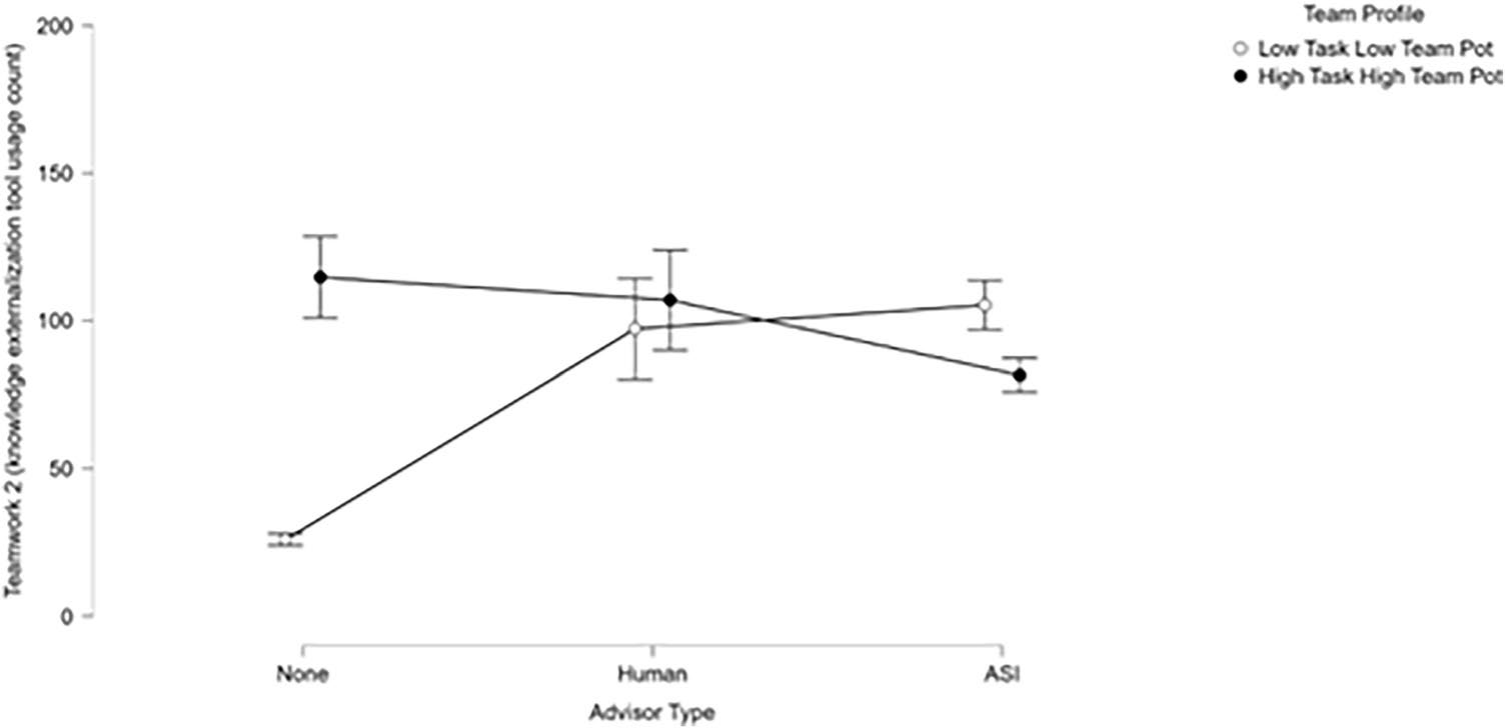
The interaction between teams’ holistic profiles and the nature of their mission advisor accounted for a large proportion of variance in teamwork measure 1 (knowledge externalization behaviors). Most importantly, it can again be observed in this visualization that ASI advisors had a notable positive effect on the behaviors of low taskwork, low teamwork potential teams such that they improved not only their task execution but also their engagement in teamwork actions. Conversely, teams that were low in taskwork and teamwork potential and had no advisor failed to employ knowledge externalization tools effectively. Teams working with a human or ASI advisor tended to employ externalization tools more efficiently to share information and achieve coordination. Those teams that were high in taskwork and teamwork potential but had no advisor tended to overuse externalization tools.

Outcomes from ANOVAs conducted to examine the main effects of team holistic profiles and advisor types on team performance measures revealed a consistent, moderately strong main effect of team holistic profile. Analysis of teamwork measure 1 (risk management failure costs) showed a strong main effect of advisory type such that teams that did not work with an advisor were performed worse at risk management (i.e., poorer at communicating threat presence knowledge). Analyses of taskwork-teamwork measure 1 and teamwork measure 2 (mission score %, knowledge externalization) both showed significant and strong interaction effects between profiles and advisor types. Over all of the mission performance measures, teams that were high in *taskwork* and *teamwork* potential performed best (or most appropriately, in the case of teamwork measure 2: knowledge externalization) by a large margin as compared to teams that were low in *taskwork* and *teamwork* potential. The effect of holistic profile was generally augmented by the presence of an advisor, and most strongly when teams worked with a human advisor. Importantly, the presence of an ASI advisor helped teams low in team and task potential as much as the human advisor when compared to the no advisor condition. Teams that were high in *taskwork* and *teamwork* potential that worked with a human advisor performed best by a moderate degree compared to most other groups, and a small margin compared to teams that were high in *taskwork* and *teamwork* that worked with an artificial social intelligence advisor (Figs. 5, 6).

## Discussion

This study set out to understand how profiling individuals on teams based on task and team potential, are predictive of team performance and how profiles differently predict team outcomes dependent on advisors who were either human, or agents imbued with artificial social intelligence. The results largely supported our primary hypothesis that individuals and teams could be profiled based on psychometric, psychographic, and brief competency test data and that higher potential on the taskwork and teamwork components of those profiles would be associated with improved performance. Our findings revealed that *teamwork* profiles were predictive of differences in measures that involved teamwork elements as well as some that were considered to involve primarily taskwork elements. These were also influential with respect to participants’ perceptions of their team and the artificial socially intelligent advisors with whom they worked. *Taskwork* profiles were also found to be predictive of some differences, though primarily on measures that were considered to be a combination of taskwork-teamwork elements. Both *taskwork* and *teamwork* profiles were found to interact to predict differences in several measures of individual and team performance as well as of perceptions of team process and ASI dependability and reasonableness.

Most importantly, our findings demonstrated that it will be critical to assign artificial, socially intelligent advisors to teams that are likely to benefit from their assistance. Inspection of the effect of advisor type in the context of low taskwork, low teamwork versus high taskwork, high teamwork potential teams revealed that only the former benefited from the assistance of ASI teams; however, the performance of the low potential teams was significantly improved by the advice of the ASIs. Relevant to selecting teams that may be appropriate for human-agent teaming, our profile analyses were able to detect differences in team behaviors as a function of their profiles and advisement. Specifically, outcomes of our analysis of the interaction between teams’ holistic profiles and the nature of the advisor with which they worked (e.g., no advisor, a human advisor, an ASI advisor) revealed that those holistic profiles were significantly and moderately-to-strongly predictive of differences in team performance. Additionally, profiles interacted with their advisor type such that profiles manifested differently when teams worked with different advisors. Particularly, teams tended to perform best when they had high potential profiles and worked with a human advisor, and worst when they had low potential profiles and did not have an advisor. Teams that had high potential profiles and worked with an ASI advisor performed near, but not quite at the level of high potential teams that worked with a human advisor. Most relevant to consideration of how human-centered artificial intelligence can improve applications, we showed how ASI improved performance for the low task – low team potential profiles. This suggests that our profiling approach can be a useful component in the development of intelligent systems because they focus on human capabilities and potential; that is, our profiles can provide artificial intelligence with information enabling a more accurate theory of mind of their human teammates.

Finally, an important contribution of this work is that we combined traditional attitudinal and perception measures found in the Human-AI literature and human-computer interaction literature with objective measures of process and performance. Further, unlike the majority of current studies on AI that use canned/scripted responses or “Wizard of Oz” setups where humans play the role of AI, our participants interacted with actual artificial intelligence. Thus, the profiling approach we developed and tested can be adapted across task domains to further research on how AI imbued with social intelligence affects both perception and process. There are some known limitations associated with this work primarily stemming from the experimental control and clarity of measures associated with a testbed that can support remote interactions between three remotely located human team members as well as an artificial socially intelligent agent. First, the USAR task and testbed represent a novel tasking environment developed by a team of SMEs to examine these and similar research questions, but they are relatively untested and not deeply explored. Ideal team behaviors in this task are unknown, as are optimal individual behaviors, and the full range of factors that may influence behaviors. Due to practical limitations in data collection, the sample size for human teams and no-advisor teams is also relatively small (only 14 each), and not all of the individuals on these teams provided complete data for profiling. Lastly, there were multiple ASIs that were paired with teams in this experiment, and each behaved somewhat differently with respect to their development of artificial theory of mind and the leveraging of that building block to assist teams. The sample size of complete data for each individual ASI is also small. As a result, some analyses that we would be interested in conducting are not feasible due to the relatively random outcomes regarding the profiles of individuals and teams that worked with each ASI.

In sum, this research examined an approach for helping artificial socially intelligent agents understand their human team members and begin to develop artificial theory of mind. We consider these to be fundamental building blocks required for the development of AI that can operate as effective teammates by attending to the social as well as the functional aspects of working on a hybrid team. Our player profiles are grounded in social science theory, and the findings presented here provide evidence that they offer meaningful insight into behavioral differences between participants. We also showed that the player profiles were useful for understanding the differences between participants’ perceptions of their team process as well as of the human and AI-enabled advisors from whom they received advice while completing missions. Further, we demonstrated that the individual player profiles can be used to construct team profiles, which are, themselves, predictive of team success with respect to overall performance as well as more nuanced dimensions such as coordination, error rate, and risk management success. Perhaps most importantly, we found that team profiles interacted with the type of advisor working with teams such that there were differences in performance, knowledge externalization behaviors, and perceptions of team process depending on whether teams worked with no advisor, a human advisor, or an artificial socially intelligent advisor. Critically, we found that ASI advisors were able to elevate performance of teams low in taskwork and teamwork potential such that they performed as well as if they had a human advisor. This suggests useful applications for both the profile approach and AI implementation in that artificial social intelligence may be best applied in situations for teams low in team and task potential. It may be worth noting that ASI advisors were always rated relatively more negatively than human advisors (note limitations), though not low relative to the provided scales. This opens up an important area of research in that there may be many reasons for this including the interaction modalities employed by advisors, the content and style of the advice provided by advisors, and because some teams performed worse when working with ASI advisors. Currently, it is unclear whether that outcome is causally linked to the ASIs, is an artifact of the interaction modality, the sample, or may be associated with differences in the missions performed by teams working with no advisor, the human advisor, or one of the ASI advisors.

### Supplementary Information

Supplementary Information 1.

Supplementary Information 2.

Supplementary Information 3.

Supplementary Information 4.

Supplementary Information 5.

Supplementary Information 6.

Supplementary Information 7.

Supplementary Information 8.

Supplementary Information 9.

Supplementary Information 10.

Supplementary Information 11.

Supplementary Information 12.

Supplementary Information 13.

Supplementary Information 14.

Supplementary Information 15.

Supplementary Information 16.

Supplementary Information 17.

Supplementary Information 18.

## Data Availability

All data used in the present manuscript may be found at 10.48349/ASU/QDQ4MH^29^. The data files employed for the analyses reported here as well as information regarding the preregistered hypotheses tested in this manuscript can be found at osf.io/t26kd ^25^.
