# Supplementary material for: Individual and team profiling to support theory of mind in artificial social intelligence
Source: Sci Rep. 2024 Jun 2;14:12635. doi: 10.1038/s41598-024-63122-8 (PMC11144695; doi:10.1038/s41598-024-63122-8)
Supplement: Supplementary file 17 — Supplementary Information 17. [file 41598_2024_63122_MOESM17_ESM.docx]

Individual and Team Profiling to Support Theory of Mind in Artificial Social Intelligence

Supplementary Information

## Table of Contents

[**Supplementary Information 1**](#_i9a9pswvar9d)

[Table of Contents 1](#_tfku28noomei)

[**Statistical Analyses 1**](#_ux3n6toipu7p)

[Individual Player Profile Analysis Details 2](#_kwcstn5iqo9e)

[Individual Profiles: Medic taskwork-teamwork metric 1 2](#_dcu8ok3ugg3t)

[Individual Profiles: Engineer taskwork metric 1 (rubble destroyed) 3](#_fxpg5e72c5kl)

[Individual Profiles: Transporter taskwork-teamwork metric 1 (critical victims moved) 3](#_qb8gm9obio7k)

[Individual Profiles: Transporter taskwork-teamwork metric 2 (critical victims evacuated) 4](#_cwzhxghd7jgd)

[Individual Profiles: Team process perceptions 5](#_fc8cvx5jcm08)

[Individual Profiles: ASI Advisor Dependable Ratings 6](#_g160a1yvot5b)

[Individual Profiles: Advisor Reasonable Ratings 7](#_esp0lxyknu29)

[Team Profile Analysis Details 8](#_1w5j1recnohz)

[Team Profiles: Team taskwork-teamwork metric 1 (mission score %) 8](#_9ark6rmr2aig)

[Team profiles: taskwork-teamwork metric 2 (heal:extract) 9](#_yia6egeoc5ii)

[Team profiles: team taskwork-teamwork metric 3 (risk management error cost) 10](#_13jk2gg60cmm)

[Team profiles: team process perception 11](#_38h4kw8zve61)

[Team profiles: ASI dependable 11](#_78ube3rb097j)

[Team profiles: ASI reasonable 12](#_gpzpoqw3bou5)

[‘Extreme’ Team Profiles X Advisor Type: taskwork-teamwork metric 1 (mission score %) 13](#_h0rrq13lmh8j)

[‘Extreme’ Team Profiles X Advisor Type: taskwork-teamwork metric 2 (risk management failure cost) 14](#_lhokqhfmm58y)

[‘Extreme’ Team Profiles X Advisor Type: teamwork metric 1 (knowledge externalization) 15](#_s54zrr2ab8ha)

##

##

## ***Statistical Analyses***

The following sections describe the analysis structure, tests, and associated descriptive statistics for the analyses presented in our report.

### ***Individual Player Profile Analysis Details***

#### ***Individual Profiles: Medic taskwork-teamwork metric 1***

Relevant data file:

SupplementaryData_IndividualPlayerProfiles

Analysis design:

Independent variable 1 = individual taskwork potential profile (low potential, high potential)

Independent variable 2 = individual teamwork potential profile (low potential, high potential)

Dependent variable = critical victims healed

Test type: two-way analysis of variance

Descriptive statistics:

Mean of Group A (high taskwork potential, high teamwork potential profile) = 11.556

Standard deviation of Group A (high taskwork potential profile, high teamwork potential profile)= 2.136

Mean of Group B (high taskwork potential profile, low teamwork potential profile)= 10.765

Standard deviation of Group B (high taskwork potential profile, low teamwork potential profile)= 2.078

Mean of Group C (low taskwork potential profile, high teamwork potential profile)= 9.806

Standard deviation of Group C (low taskwork potential profile, high teamwork potential profile) = 2.400

Mean of Group D (low taskwork potential profile, low teamwork potential profile) = 10.00

Standard deviation of Group D (low taskwork potential profile, low teamwork potential profile) =2.299

Levene’s test for homogeneity of variances:

F-statistic = 0.661

df1 = 3

df2 = 105

p value= .578

ANOVA results for team taskwork potential profile:

F-statistic (degrees of freedom = 1,105) = 7.815

p-value = .006

Alpha level = .05

partial eta-squared (η²) = 0.069

ANOVA results for team teamwork potential profile:

F-statistic (degrees of freedom = 1,105) = 0.440

p-value = 0.509

Alpha level = .05

partial eta-squared (η²) = 0.004

ANOVA results for team taskwork potential profile & team teamwork potential profile interaction:

F-statistic (degrees of freedom = 1,105) = 1.200

p-value = 0.276

Alpha level =.05

partial eta-squared (η²) = 0.011

#### ***Individual Profiles: Engineer taskwork metric 1 (rubble destroyed)***

Relevant data file:

SupplementaryData_IndividualPlayerProfiles

Analysis design:

Independent variable 1 = individual taskwork potential profile (low potential, high potential)

Independent variable 2 = individual teamwork potential profile (low potential, high potential)

Dependent variable = rubble destroyed

Test type: two-way analysis of variance

Descriptive statistics:

Mean of Group A (high taskwork potential, high teamwork potential profile) = 10.966

Standard deviation of Group A (high taskwork potential profile, high teamwork potential profile)= 2.322

Mean of Group B (high taskwork potential profile, low teamwork potential profile)= 10.565

Standard deviation of Group B (high taskwork potential profile, low teamwork potential profile)= 1.701

Mean of Group C (low taskwork potential profile, high teamwork potential profile)= 10.088

Standard deviation of Group C (low taskwork potential profile, high teamwork potential profile) = 2.756

Mean of Group D (low taskwork potential profile, low teamwork potential profile) = 10.714

Standard deviation of Group D (low taskwork potential profile, low teamwork potential profile) =2.053

Levene’s test for homogeneity of variances:

F-statistic = 5.424

df1 = 3.00

df2 = 103.00

p value= .002

ANOVA results for team taskwork potential profile:

F-statistic (degrees of freedom = 1,105) = 0.641

p-value = .425

Alpha level = .05

partial eta-squared (η²) = .006

ANOVA results for team teamwork potential profile:

F-statistic (degrees of freedom = 1,105) = 0.062

p-value = .804

Alpha level = .05

partial eta-squared (η²) = .0006

ANOVA results for team taskwork potential profile & team teamwork potential profile interaction:

F-statistic (degrees of freedom = 1,105) = 1.274

p-value = .262

Alpha level = .05

partial eta-squared (η²) = 0.012

#### ***Individual Profiles: Transporter taskwork-teamwork metric 1 (critical victims moved)***

Relevant data file:

SupplementaryData_IndividualPlayerProfiles

Analysis design:

Independent variable 1 = individual taskwork potential profile (low potential, high potential)

Independent variable 2 = individual teamwork potential profile (low potential, high potential)

Dependent variable = transporter critical victims moved

Test type: two-way analysis of variance

Descriptive statistics:

Mean of Group A (high taskwork potential, high teamwork potential profile) = 6.448

Standard deviation of Group A (high taskwork potential profile, high teamwork potential profile)= 3.511

Mean of Group B (high taskwork potential profile, low teamwork potential profile)= 4.500

Standard deviation of Group B (high taskwork potential profile, low teamwork potential profile)= 2.724

Mean of Group C (low taskwork potential profile, high teamwork potential profile)= 5.500

Standard deviation of Group C (low taskwork potential profile, high teamwork potential profile) = 2.700

Mean of Group D (low taskwork potential profile, low teamwork potential profile) = 4.500

Standard deviation of Group D (low taskwork potential profile, low teamwork potential profile) =3.677

Levene’s test for homogeneity of variances:

F-statistic = 0.822

df1 = 3

df2 = 105

p value= .485

ANOVA results for team taskwork potential profile:

F-statistic (degrees of freedom = 1,105) =0.579

p-value = .448

Alpha level = .05

partial eta-squared (η²) =.005

ANOVA results for team teamwork potential profile:

F-statistic (degrees of freedom = 1,105) = 5.595

p-value = .020

Alpha level = .05

partial eta-squared (η²) = .051

ANOVA results for team taskwork potential profile & team teamwork potential profile interaction:

F-statistic (degrees of freedom = 1,105) = .579

p-value = .448

Alpha level = .05

partial eta-squared (η²) = .005

#### ***Individual Profiles: Transporter taskwork-teamwork metric 2 (critical victims evacuated)***

Relevant data file:

SupplementaryData_IndividualPlayerProfiles

Analysis design:

Independent variable 1 = individual taskwork potential profile (low potential, high potential)

Independent variable 2 = individual teamwork potential profile (low potential, high potential)

Dependent variable = transporter critical victims evacuated

Descriptive statistics:

Mean of Group A (high taskwork potential, high teamwork potential profile) = 4

Standard deviation of Group A (high taskwork potential profile, high teamwork potential profile)=1.711

Mean of Group B (high taskwork potential profile, low teamwork potential profile)= 3.25

Standard deviation of Group B (high taskwork potential profile, low teamwork potential profile)= 1.713

Mean of Group C (low taskwork potential profile, high teamwork potential profile)= 2.451

Standard deviation of Group C (low taskwork potential profile, high teamwork potential profile) = 0.433

Mean of Group D (low taskwork potential profile, low teamwork potential profile) = 2.964

Standard deviation of Group D (low taskwork potential profile, low teamwork potential profile) =2.186

Levene’s test for homogeneity of variances:

F-statistic = 1.811

df1 = 3

df2 = 105

p value= .150

ANOVA results for team taskwork potential profile:

F-statistic (degrees of freedom = 1,105) = 0.299

p-value = .586

Alpha level = .05

partial eta-squared (η²) = .003

ANOVA results for team teamwork potential profile:

F-statistic (degrees of freedom = 1,105) = 4.064

p-value = 0.046

Alpha level = .05

partial eta-squared (η²) = .037

ANOVA results for team taskwork potential profile & team teamwork potential profile interaction:

F-statistic (degrees of freedom = 1,105) = .026

p-value = .873

Alpha level = .05

partial eta-squared (η²) = .0002

#### ***Individual Profiles: Team process perceptions***

Relevant data file:

SupplementaryData_IndividualPlayerProfiles

Analysis design:

Independent variable 1 = individual taskwork potential profile (low potential, high potential)

Independent variable 2 = individual teamwork potential profile (low potential, high potential)

Dependent variable = players’ team process average rating

Descriptive statistics:

Mean of Group A (high taskwork potential, high teamwork potential profile) = 4.561

Standard deviation of Group A (high taskwork potential profile, high teamwork potential profile)=0.453

Mean of Group B (high taskwork potential profile, low teamwork potential profile)= 4.405

Standard deviation of Group B (high taskwork potential profile, low teamwork potential profile)= 0.495

Mean of Group C (low taskwork potential profile, high teamwork potential profile)= 4.443

Standard deviation of Group C (low taskwork potential profile, high teamwork potential profile) = 0.597

Mean of Group D (low taskwork potential profile, low teamwork potential profile) = 4.211

Standard deviation of Group D (low taskwork potential profile, low teamwork potential profile) = 0.630

Levene’s test for homogeneity of variances:

F-statistic = 4.227

df1 = 3

df2 = 253

p value= .006

ANOVA results for team taskwork potential profile:

F-statistic (degrees of freedom = 1,105) = 6.172

p-value = .013

Alpha level = .05

partial eta-squared (η²) = .019

ANOVA results for team teamwork potential profile:

F-statistic (degrees of freedom = 1,105) = 9.596

p-value = .002

Alpha level = .05

partial eta-squared (η²) = .029

ANOVA results for team taskwork potential profile & team teamwork potential profile interaction:

F-statistic (degrees of freedom = 1,105) = 0.374

p-value = .541

Alpha level = .05

partial eta-squared (η²) = .001

#### ***Individual Profiles: ASI Advisor Dependable Ratings***

Relevant data file:

SupplementaryData_IndividualPlayerProfiles_ASIonly

Analysis design:

Independent variable 1 = individual taskwork potential profile (low potential, high potential)

Independent variable 2 = individual teamwork potential profile (low potential, high potential)

Dependent variable = player ratings of whether advisor was dependable

Test type: two-way analysis of variance

Descriptive statistics:

Mean of Group A (high taskwork potential, high teamwork potential profile) = 4.645

Standard deviation of Group A (high taskwork potential profile, high teamwork potential profile)= 1.847

Mean of Group B (high taskwork potential profile, low teamwork potential profile)= 4.735

Standard deviation of Group B (high taskwork potential profile, low teamwork potential profile)= 1.868

Mean of Group C (low taskwork potential profile, high teamwork potential profile)= 4.793

Standard deviation of Group C (low taskwork potential profile, high teamwork potential profile) = 1.910

Mean of Group D (low taskwork potential profile, low teamwork potential profile) = 4.203

Standard deviation of Group D (low taskwork potential profile, low teamwork potential profile) = 1.747

Levene’s test for homogeneity of variances:

F-statistic = 1.251

df1 = 3

df2 = 253

p value= .292

ANOVA results for team taskwork potential profile:

F-statistic (degrees of freedom = 1,253) = .671

p-value = .413

Alpha level = .05

partial eta-squared (η²) = .003

ANOVA results for team teamwork potential profile:

F-statistic (degrees of freedom = 1,253) = 1.138

p-value = .287

Alpha level = .05

partial eta-squared (η²) = .004

ANOVA results for team taskwork potential profile & team teamwork potential profile interaction:

F-statistic (degrees of freedom = 1,253) =2.099

p-value = .149

Alpha level = .05

partial eta-squared (η²) = .008

#### ***Individual Profiles: Advisor Reasonable Ratings***

Relevant data file:

SupplementaryData_IndividualPlayerProfiles_ASIonly

Analysis design:

Independent variable 1 = individual taskwork potential profile (low potential, high potential)

Independent variable 2 = individual teamwork potential profile (low potential, high potential)

Dependent variable = advisor rating: suggestions reasonable

Descriptive statistics:

Mean of Group A (high taskwork potential, high teamwork potential profile) = 5.23

Standard deviation of Group A (high taskwork potential profile, high teamwork potential profile)=1.641

Mean of Group B (high taskwork potential profile, low teamwork potential profile)= 5.673

Standard deviation of Group B (high taskwork potential profile, low teamwork potential profile)= 1.533

Mean of Group C (low taskwork potential profile, high teamwork potential profile)= 5.695

Standard deviation of Group C (low taskwork potential profile, high teamwork potential profile) = 1.385

Mean of Group D (low taskwork potential profile, low teamwork potential profile) = 4.969

Standard deviation of Group D (low taskwork potential profile, low teamwork potential profile) =1.759

Levene’s test for homogeneity of variances:

F-statistic = 1.475

df1 = 3

df2 = 253

p value= .222

ANOVA results for team taskwork potential profile:

F-statistic (degrees of freedom = 1,105) = 0.356

p-value = .551

Alpha level = .05

partial eta-squared (η²) = .001

ANOVA results for team teamwork potential profile:

F-statistic (degrees of freedom = 1,105) = 0.497

p-value = 0.482

Alpha level = .05

partial eta-squared (η²) = .002

ANOVA results for team taskwork potential profile & team teamwork potential profile interaction:

F-statistic (degrees of freedom = 1,105) = 8.552

p-value = .004

Alpha level = .05

partial eta-squared (η²) = .033

### ***Team Profile Analysis Details***

#### ***Team Profiles: Team taskwork-teamwork metric 1 (mission score %)***

Relevant data file:

SupplementaryData_TeamProfiles

Analysis design:

Independent variable 1 = team taskwork potential profile (low potential, high potential)

Independent variable 2 = team teamwork potential profile (low potential, high potential)

Dependent variable = mission score percentage

Descriptive statistics:

Mean of Group A (high taskwork potential, high teamwork potential profile) = 69.368

Standard deviation of Group A (high taskwork potential profile, high teamwork potential profile)= 16.086

Mean of Group B (high taskwork potential profile, low teamwork potential profile)= 63.801

Standard deviation of Group B (high taskwork potential profile, low teamwork potential profile)= 12.361

Mean of Group C (low taskwork potential profile, high teamwork potential profile)= 56.096

Standard deviation of Group C (low taskwork potential profile, high teamwork potential profile) = 15.783

Mean of Group D (low taskwork potential profile, low teamwork potential profile) = 65.675

Standard deviation of Group D (low taskwork potential profile, low teamwork potential profile) = 12.571

Levene’s test for homogeneity of variances:

F-statistic = 1.130

df1 = 3

df2 = 91

p value= .341

ANOVA results for team teamwork potential profile:

F-statistic (degrees of freedom = 1,91) =0.436

p-value = 0.511

Alpha level = .05

partial eta-squared (η²) = .005

ANOVA results for team taskwork potential profile:

F-statistic (degrees of freedom = 1,91) = 3.518

p-value = 0.064

Alpha level = .05

partial eta-squared (η²) = .037

ANOVA results for team teamwork potential profile & team taskwork potential profile interaction:

F-statistic (degrees of freedom = 1,91) = 6.212

p-value = .014

Alpha level = .05

partial eta-squared (η²) = .064

#### ***Team profiles: taskwork-teamwork metric 2 (heal:extract)***

Relevant data file:

SupplementaryData_Teamprofiles

Analysis design:

Independent variable 1 = team taskwork potential profile (low potential, high potential)

Independent variable 2 = team teamwork potential profile (low potential, high potential)

Dependent variable = team’s heal-to-extract error rate

Descriptive statistics:

Mean of Group A (high taskwork potential, high teamwork potential profile) = 0.194

Standard deviation of Group A (high taskwork potential profile, high teamwork potential profile)= 0.119

Mean of Group B (high taskwork potential profile, low teamwork potential profile)= 0.267

Standard deviation of Group B (high taskwork potential profile, low teamwork potential profile)= 0.128

Mean of Group C (low taskwork potential profile, high teamwork potential profile)= 0.253

Standard deviation of Group C (low taskwork potential profile, high teamwork potential profile) = 0.143

Mean of Group D (low taskwork potential profile, low teamwork potential profile) = 0.216

Standard deviation of Group D (low taskwork potential profile, low teamwork potential profile) = 0.130

Levene’s test for homogeneity of variances:

F-statistic = 0.423

df1 = 3

df2 = 91

p value= .737

ANOVA results for team taskwork potential profile:

F-statistic (degrees of freedom = 1,91) =.025

p-value = .875

Alpha level = .05

partial eta-squared (η²) = .0002

ANOVA results for team teamwork potential profile:

F-statistic (degrees of freedom = 1,91) = .473

p-value = .493

Alpha level = .05

partial eta-squared (η²) =.005

ANOVA results for team taskwork potential profile & team teamwork potential profile interaction:

F-statistic (degrees of freedom = 1,91) = 4.120

p-value = .045

Alpha level = .05

partial eta-squared (η²) = .043

#### ***Team profiles: team taskwork-teamwork metric 3 (risk management error cost)***

Relevant data file:

SupplementaryData_TeamProfiles

Analysis design:

Independent variable 1 = team taskwork potential profile (low potential, high potential)

Independent variable 2 = team teamwork potential profile (low potential, high potential)

Dependent variable = team’s time cost for risk management errors

Descriptive statistics:

Mean of Group A (high taskwork potential, high teamwork potential profile) = 202749.759

Standard deviation of Group A (high taskwork potential profile, high teamwork potential profile)= 67214.989

Mean of Group B (high taskwork potential profile, low teamwork potential profile)= 281527.389

Standard deviation of Group B (high taskwork potential profile, low teamwork potential profile)= 127907.141

Mean of Group C (low taskwork potential profile, high teamwork potential profile)= 289662.0

Standard deviation of Group C (low taskwork potential profile, high teamwork potential profile) = 169480.146

Mean of Group D (low taskwork potential profile, low teamwork potential profile) = 219368.870

Standard deviation of Group D (low taskwork potential profile, low teamwork potential profile) = 107678.771

Levene’s test for homogeneity of variances:

F-statistic = 3.734

df1 = 3

df2 = 90

p value= 0.014

ANOVA results for team taskwork potential profile:

F-statistic (degrees of freedom = 1,90) = 0.238

p-value = 0.627

Alpha level = .05

partial eta-squared (η²) = .003

ANOVA results for team teamwork potential profile:

F-statistic (degrees of freedom = 1,90) =0.028

p-value = 0.867

Alpha level = .05

partial eta-squared (η²) = .0003

ANOVA results for team taskwork potential profile & team teamwork potential profile interaction:

F-statistic (degrees of freedom = 1,90) = 8.647

p-value = .004

Alpha level = .05

partial eta-squared (η²) = .088

#### ***Team profiles: team process perception***

Relevant data file:

SupplementaryData_TeamProfiles_ASIonly

Analysis design:

Independent variable 1 = team taskwork potential profile (low potential, high potential)

Independent variable 2 = team teamwork potential profile (low potential, high potential)

Dependent variable = team’s average team process rating

Descriptive statistics:

Mean of Group A (high taskwork potential, high teamwork potential profile) = 4.566

Standard deviation of Group A (high taskwork potential profile, high teamwork potential profile)= 0.248

Mean of Group B (high taskwork potential profile, low teamwork potential profile)= 4.306

Standard deviation of Group B (high taskwork potential profile, low teamwork potential profile)= 0.279

Mean of Group C (low taskwork potential profile, high teamwork potential profile)= 4.288

Standard deviation of Group C (low taskwork potential profile, high teamwork potential profile) = 0.573

Mean of Group D (low taskwork potential profile, low teamwork potential profile) = 4.464

Standard deviation of Group D (low taskwork potential profile, low teamwork potential profile) = 0.304

Levene’s test for homogeneity of variances:

F-statistic = 7.136

df1 = 3

df2 = 63

p value= <.001

ANOVA results for team taskwork potential profile:

F-statistic (degrees of freedom = 1,63) = .015

p-value = 0.902

Alpha level = .05

partial eta-squared (η²) =.0002

ANOVA results for team teamwork potential profile:

F-statistic (degrees of freedom = 1,63) = 0.834

p-value = .365

Alpha level = .05

partial eta-squared (η²) = .013

ANOVA results for team taskwork potential profile & team teamwork potential profile interaction:

F-statistic (degrees of freedom = 1,63) = 6.340

p-value = .014

Alpha level = .05

partial eta-squared (η²) = .091

#### ***Team profiles: ASI dependable***

Relevant data file:

SupplementaryData_TeamProfiles_ASIonly

Analysis design:

Independent variable 1 = team taskwork potential profile (low potential, high potential)

Independent variable 2 = team teamwork potential profile (low potential, high potential)

Dependent variable = team’s rating of whether ASI was dependable

Descriptive statistics:

Mean of Group A (high taskwork potential, high teamwork potential profile) = 4.284

Standard deviation of Group A (high taskwork potential profile, high teamwork potential profile)= 1.471

Mean of Group B (high taskwork potential profile, low teamwork potential profile)= 4.417

Standard deviation of Group B (high taskwork potential profile, low teamwork potential profile)= 1.111

Mean of Group C (low taskwork potential profile, high teamwork potential profile)= 4.651

Standard deviation of Group C (low taskwork potential profile, high teamwork potential profile) = 1.284

Mean of Group D (low taskwork potential profile, low teamwork potential profile) = 3.912

Standard deviation of Group D (low taskwork potential profile, low teamwork potential profile) = 0.936

Levene’s test for homogeneity of variances:

F-statistic = 1.823

df1 = 3

df2 = 63

p value= .152

ANOVA results for team taskwork potential profile:

F-statistic (degrees of freedom = 1,63) = 0.051

p-value = .822

Alpha level = .05

partial eta-squared (η²) = .0008

ANOVA results for team teamwork potential profile:

F-statistic (degrees of freedom = 1,63) = 0.980

p-value = .326

Alpha level = .05

partial eta-squared (η²) = .015

ANOVA results for team taskwork potential profile & team teamwork potential profile interaction:

F-statistic (degrees of freedom = 1,63) = 2.022

p-value = .160

Alpha level = .05

partial eta-squared (η²) = .031

#### ***Team profiles: ASI reasonable***

Relevant data file:

SupplementaryData_TeamProfiles_ASIonly

Analysis design:

Independent variable 1 = team taskwork potential profile (low potential, high potential)

Independent variable 2 = team teamwork potential profile (low potential, high potential)

Dependent variable = team’s rating of whether ASI was reasonable

Descriptive statistics:

Mean of Group A (high taskwork potential, high teamwork potential profile) = 4.804

Standard deviation of Group A (high taskwork potential profile, high teamwork potential profile)= 1.533

Mean of Group B (high taskwork potential profile, low teamwork potential profile)= 5.861

Standard deviation of Group B (high taskwork potential profile, low teamwork potential profile)= 0.926

Mean of Group C (low taskwork potential profile, high teamwork potential profile)= 5.349

Standard deviation of Group C (low taskwork potential profile, high teamwork potential profile) = 0.920

Mean of Group D (low taskwork potential profile, low teamwork potential profile) = 4.951

Standard deviation of Group D (low taskwork potential profile, low teamwork potential profile) = 0.935

Levene’s test for homogeneity of variances:

F-statistic = 3.522

df1 = 3

df2 = 63

p value= .020

ANOVA results for team taskwork potential profile:

F-statistic (degrees of freedom = 1,63) = 0.433

p-value = .513

Alpha level = .05

partial eta-squared (η²) = .007

ANOVA results for team teamwork potential profile:

F-statistic (degrees of freedom = 1,63) = 1.411

p-value = .239

Alpha level = .05

partial eta-squared (η²) = .022

ANOVA results for team taskwork potential profile & team teamwork potential profile interaction:

F-statistic (degrees of freedom = 1,63) = 6.883

p-value = .011

Alpha level = .05

partial eta-squared (η²) = 0.098

#### ***‘Extreme’ Team Profiles X Advisor Type: taskwork-teamwork metric 1 (mission score %)***

Relevant data file:

SupplementaryData_ExtremeTeamProfiles

Analysis design:

Independent variable 1 = team holistic profile (low taskwork low teamwork, high taskwork high teamwork)

Independent variable 2 = advisor type (no advisor, human advisor, ASI advisor)

Dependent variable = team’s mission score percentage

Descriptive statistics:

Mean of Group A (ASI advisor, high teamwork high taskwork) = 61.858

Standard deviation of Group A (ASI advisor, high teamwork high taskwork)= 16.918

Mean of Group B (ASI advisor, low teamwork low taskwork) = 67.554

Standard deviation of Group B (ASI advisor, low teamwork low taskwork)= 13.185

Mean of Group C (Human advisor, high teamwork high taskwork) = 79.368

Standard deviation of Group C (Human advisor, high teamwork high taskwork)= 2.844

Mean of Group D (Human advisor, low teamwork low taskwork) = 62.895

Standard deviation of Group D (Human advisor, low teamwork low taskwork)= 11.284

Mean of Group E (No advisor, high teamwork high taskwork) = 79.079

Standard deviation of Group E (No advisor, high teamwork high taskwork) = 9.849

Mean of Group F (No advisor, low teamwork low taskwork) = 55.263

Standard deviation of Group E (No advisor, low teamwork low taskwork) = 2.233

Levene’s test for homogeneity of variances:

F-statistic = 3.262

df1 = 5

df2 = 47

p value= .013

ANOVA results for advisor type:

F-statistic (degrees of freedom = 2,47) = .828

p-value = .443

Alpha level = .05

partial eta-squared (η²) = .034

ANOVA results for team profile:

F-statistic (degrees of freedom = 1,47) = 5.574

p-value = .022

Alpha level = .05

partial eta-squared (η²) =.106

ANOVA results for profile X advisor type interaction:

F-statistic (degrees of freedom = 2,47) = 4.807

p-value = .013

Alpha level = .05

partial eta-squared (η²) = .170

#### ***‘Extreme’ Team Profiles X Advisor Type: taskwork-teamwork metric 2 (risk management failure cost)***

Relevant data file:

SupplementaryData_ExtremeTeamProfiles

Analysis design:

Independent variable 1 = team holistic profile (low taskwork low teamwork, high taskwork high teamwork)

Independent variable 2 = advisor type (no advisor, human advisor, ASI advisor)

Dependent variable = risk management failure cost [ms]

Descriptive statistics:

Mean of Group A (ASI advisor, high teamwork high taskwork) = 190467.188

Standard deviation of Group A (ASI advisor, high teamwork high taskwork)= 63597.949

Mean of Group B (ASI advisor, low teamwork low taskwork) = 202606.882

Standard deviation of Group B (ASI advisor, low teamwork low taskwork)= 98504.867

Mean of Group C (Human advisor, high teamwork high taskwork) = 185043.4

Standard deviation of Group C (Human advisor, high teamwork high taskwork)= 83386.464

Mean of Group D (Human advisor, low teamwork low taskwork) = 204497

Standard deviation of Group D (Human advisor, low teamwork low taskwork)= 72754.624

Mean of Group E (No advisor, high teamwork high taskwork) = 238381.375

Standard deviation of Group E (No advisor, high teamwork high taskwork) = 65627.625

Mean of Group F (No advisor, low teamwork low taskwork) = 391589.5

Standard deviation of Group E (No advisor, low teamwork low taskwork) = 137738.037

Levene’s test for homogeneity of variances:

F-statistic = 0.635

df1 = 5

df2 = 46

p value= .674

ANOVA results for advisor type:

F-statistic (degrees of freedom = 2,47) = 5.962

p-value = .005

Alpha level = .05

partial eta-squared (η²) = .206

ANOVA results for team profile:

F-statistic (degrees of freedom = 1,47) = 5.962

p-value = .043

Alpha level = .05

partial eta-squared (η²) =.086

ANOVA results for profile X advisor type interaction:

F-statistic (degrees of freedom = 2,47) = 2.061

p-value = .139

Alpha level = .05

partial eta-squared (η²) = .082

#### ***‘Extreme’ Team Profiles X Advisor Type: teamwork metric 1 (knowledge externalization)***

Relevant data file:

SupplementaryData_ExtremeTeamProfiles

Analysis design:

Independent variable 1 = team holistic profile (low taskwork low teamwork, high taskwork high teamwork)

Independent variable 2 = advisor type (no advisor, human advisor, ASI advisor)

Dependent variable = team’s usage of knowledge externalization tools

Descriptive statistics:

Mean of Group A (ASI advisor, high teamwork high taskwork) = 81.588

Standard deviation of Group A (ASI advisor, high teamwork high taskwork)= 23.773

Mean of Group B (ASI advisor, low teamwork low taskwork) = 109.059

Standard deviation of Group B (ASI advisor, low teamwork low taskwork)= 32.7

Mean of Group C (Human advisor, high teamwork high taskwork) = 107

Standard deviation of Group C (Human advisor, high teamwork high taskwork)= 37.855

Mean of Group D (Human advisor, low teamwork low taskwork) = 97.250

Standard deviation of Group D (Human advisor, low teamwork low taskwork)= 34.131

Mean of Group E (No advisor, high teamwork high taskwork) = 114.750

Standard deviation of Group E (No advisor, high teamwork high taskwork) = 39.008

Mean of Group F (No advisor, low teamwork low taskwork) = 26

Standard deviation of Group E (No advisor, low teamwork low taskwork) = 2.828

Levene’s test for homogeneity of variances:

F-statistic = 0.825

df1 = 5

df2 = 47

p value= 0.538

ANOVA results for advisor type:

F-statistic (degrees of freedom = 2,47) = 2.141

p-value = 0.129

Alpha level = .05

partial eta-squared (η²) = .084

ANOVA results for team profile:

F-statistic (degrees of freedom = 1,47) = 4.319

p-value = .043

Alpha level = .05

partial eta-squared (η²) = .084

ANOVA results for team profile X advisor type:

F-statistic (degrees of freedom = 2,47) = 9.613

p-value = <.001

Alpha level = .05

partial eta-squared (η²) = .290
