# Supplementary material for: Individual and team profiling to support theory of mind in artificial social intelligence
Source: Sci Rep. 2024 Jun 2;14:12635. doi: 10.1038/s41598-024-63122-8 (PMC11144695; doi:10.1038/s41598-024-63122-8)
Supplement: Supplementary file 18 — Supplementary Information 18. [file 41598_2024_63122_MOESM18_ESM.pdf]

Individual and Team Profiling to Support Theory of Mind in Artificial Social Intelligence

Supplementary Information

Table of Contents

Supplementary Information....1
Table of Contents....1
Statistical Analyses....1
Individual Player Profile Analysis Details....2
Individual Profiles: Medic taskwork-teamwork metric 1....2
Individual Profiles: Engineer taskwork metric 1 (rubble destroyed)....3
Individual Profiles: Transporter taskwork-teamwork metric 1 (critical victims moved)....3
Individual Profiles: Transporter taskwork-teamwork metric 2 (critical victims evacuated)....4
Individual Profiles: Team process perceptions....5
Individual Profiles: ASI Advisor Dependable Ratings....6
Individual Profiles: Advisor Reasonable Ratings....7
Team Profile Analysis Details....8
Team Profiles: Team taskwork-teamwork metric 1 (mission score %)....8
Team profiles: taskwork-teamwork metric 2 (heal:extract)....9
Team profiles: team taskwork-teamwork metric 3 (risk management error cost)....10
Team profiles: team process perception....11
Team profiles: ASI dependable....11
Team profiles: ASI reasonable....12
'Extreme' Team Profiles X Advisor Type: taskwork-teamwork metric 1 (mission score %)....13
'Extreme' Team Profiles X Advisor Type: taskwork-teamwork metric 2 (risk management failure cost)....14
'Extreme' Team Profiles X Advisor Type: teamwork metric 1 (knowledge externalization)....15

Statistical Analyses

The following sections describe the analysis structure, tests, and associated descriptive statistics for the analyses presented in our report.

Individual Player Profile Analysis Details

Individual Profiles: Medic taskwork-teamwork metric 1

Relevant data file:
SupplementaryData_IndividualPlayerProfiles

Analysis design:
Independent variable 1 = individual taskwork potential profile (low potential, high potential)
Independent variable 2 = individual teamwork potential profile (low potential, high potential)
Dependent variable = critical victims healed

Test type: two-way analysis of variance

Descriptive statistics:
Mean of Group A (high taskwork potential, high teamwork potential profile) = 11.556
Standard deviation of Group A (high taskwork potential profile, high teamwork potential profile)= 2.136

Mean of Group B (high taskwork potential profile, low teamwork potential profile)= 10.765
Standard deviation of Group B (high taskwork potential profile, low teamwork potential profile)= 2.078

Mean of Group C (low taskwork potential profile, high teamwork potential profile)= 9.806
Standard deviation of Group C (low taskwork potential profile, high teamwork potential profile) = 2.400

Mean of Group D (low taskwork potential profile, low teamwork potential profile) = 10.00
Standard deviation of Group D (low taskwork potential profile, low teamwork potential profile) =2.299

Levene’s test for homogeneity of variances:
F-statistic = 0.661
df1 = 3
df2 = 105
p value= .578

ANOVA results for team taskwork potential profile:
F-statistic (degrees of freedom = 1,105) = 7.815
p-value = .006
Alpha level = .05
partial eta-squared (η^2) = 0.069

ANOVA results for team teamwork potential profile:
F-statistic (degrees of freedom = 1,105) = 0.440
p-value = 0.509
Alpha level = .05
partial eta-squared (η^2) = 0.004

ANOVA results for team taskwork potential profile & team teamwork potential profile interaction:
F-statistic (degrees of freedom = 1,105) = 1.200
p-value = 0.276

Alpha level =.05
partial eta-squared (η^2) = 0.011

Individual Profiles: Engineer taskwork metric 1 (rubble destroyed)

Relevant data file:
SupplementaryData_IndividualPlayerProfiles

Analysis design:
Independent variable 1 = individual taskwork potential profile (low potential, high potential)
Independent variable 2 = individual teamwork potential profile (low potential, high potential)
Dependent variable = rubble destroyed

Test type: two-way analysis of variance

Descriptive statistics:
Mean of Group A (high taskwork potential, high teamwork potential profile) = 10.966
Standard deviation of Group A (high taskwork potential profile, high teamwork potential profile)= 2.322

Mean of Group B (high taskwork potential profile, low teamwork potential profile)= 10.565
Standard deviation of Group B (high taskwork potential profile, low teamwork potential profile)= 1.701

Mean of Group C (low taskwork potential profile, high teamwork potential profile)= 10.088
Standard deviation of Group C (low taskwork potential profile, high teamwork potential profile) = 2.756

Mean of Group D (low taskwork potential profile, low teamwork potential profile) = 10.714
Standard deviation of Group D (low taskwork potential profile, low teamwork potential profile) =2.053

Levene's test for homogeneity of variances:
F-statistic = 5.424
df1 = 3.00
df2 = 103.00
p value= .002

ANOVA results for team taskwork potential profile:
F-statistic (degrees of freedom = 1,105) = 0.641
p-value = .425
Alpha level = .05
partial eta-squared (η^2) = .006

ANOVA results for team teamwork potential profile:
F-statistic (degrees of freedom = 1,105) = 0.062
p-value = .804
Alpha level = .05
partial eta-squared (η^2) = .0006

ANOVA results for team taskwork potential profile & team teamwork potential profile interaction:
F-statistic (degrees of freedom = 1,105) = 1.274
p-value = .262
Alpha level = .05
partial eta-squared (η^2) = 0.012

Individual Profiles: Transporter taskwork-teamwork metric 1 (critical victims moved)

Relevant data file:
SupplementaryData_IndividualPlayerProfiles

Analysis design:
Independent variable 1 = individual taskwork potential profile (low potential, high potential)
Independent variable 2 = individual teamwork potential profile (low potential, high potential)
Dependent variable = transporter critical victims moved

Test type: two-way analysis of variance

Descriptive statistics:
Mean of Group A (high taskwork potential, high teamwork potential profile) = 6.448
Standard deviation of Group A (high taskwork potential profile, high teamwork potential profile)= 3.511

Mean of Group B (high taskwork potential profile, low teamwork potential profile)= 4.500
Standard deviation of Group B (high taskwork potential profile, low teamwork potential profile)= 2.724

Mean of Group C (low taskwork potential profile, high teamwork potential profile)= 5.500
Standard deviation of Group C (low taskwork potential profile, high teamwork potential profile) = 2.700

Mean of Group D (low taskwork potential profile, low teamwork potential profile) = 4.500
Standard deviation of Group D (low taskwork potential profile, low teamwork potential profile) =3.677

Levene's test for homogeneity of variances:
F-statistic = 0.822
df1 = 3
df2 = 105
p value= .485

ANOVA results for team taskwork potential profile:
F-statistic (degrees of freedom = 1,105) =0.579
p-value = .448
Alpha level = .05
partial eta-squared (η^2) =.005

ANOVA results for team teamwork potential profile:
F-statistic (degrees of freedom = 1,105) = 5.595
p-value = .020
Alpha level = .05
partial eta-squared (η^2) = .051

ANOVA results for team taskwork potential profile & team teamwork potential profile interaction:
F-statistic (degrees of freedom = 1,105) = .579
p-value = .448
Alpha level = .05
partial eta-squared (η^2) = .005

Individual Profiles: Transporter taskwork-teamwork metric 2 (critical victims evacuated)

Relevant data file:
SupplementaryData_IndividualPlayerProfiles

Analysis design:
Independent variable 1 = individual taskwork potential profile (low potential, high potential)
Independent variable 2 = individual teamwork potential profile (low potential, high potential)
Dependent variable = transporter critical victims evacuated

Descriptive statistics:
Mean of Group A (high taskwork potential, high teamwork potential profile) = 4
Standard deviation of Group A (high taskwork potential profile, high teamwork potential profile)=1.711

Mean of Group B (high taskwork potential profile, low teamwork potential profile)= 3.25
Standard deviation of Group B (high taskwork potential profile, low teamwork potential profile)= 1.713

Mean of Group C (low taskwork potential profile, high teamwork potential profile)= 2.451
Standard deviation of Group C (low taskwork potential profile, high teamwork potential profile) = 0.433

Mean of Group D (low taskwork potential profile, low teamwork potential profile) = 2.964
Standard deviation of Group D (low taskwork potential profile, low teamwork potential profile) =2.186

Levene's test for homogeneity of variances:
F-statistic = 1.811
df1 = 3
df2 = 105
p value= .150

ANOVA results for team taskwork potential profile:
F-statistic (degrees of freedom = 1,105) = 0.299
p-value = .586
Alpha level = .05
partial eta-squared (η^2) = .003

ANOVA results for team teamwork potential profile:
F-statistic (degrees of freedom = 1,105) = 4.064
p-value = 0.046
Alpha level = .05
partial eta-squared (η^2) = .037

ANOVA results for team taskwork potential profile & team teamwork potential profile interaction:
F-statistic (degrees of freedom = 1,105) = .026
p-value = .873
Alpha level = .05
partial eta-squared (η^2) = .0002

Individual Profiles: Team process perceptions

Relevant data file:
SupplementaryData_IndividualPlayerProfiles

Analysis design:
Independent variable 1 = individual taskwork potential profile (low potential, high potential)
Independent variable 2 = individual teamwork potential profile (low potential, high potential)
Dependent variable = players' team process average rating

Descriptive statistics:

Mean of Group A (high taskwork potential, high teamwork potential profile) = 4.561
Standard deviation of Group A (high taskwork potential profile, high teamwork potential profile)=0.453

Mean of Group B (high taskwork potential profile, low teamwork potential profile)= 4.405
Standard deviation of Group B (high taskwork potential profile, low teamwork potential profile)= 0.495

Mean of Group C (low taskwork potential profile, high teamwork potential profile)= 4.443
Standard deviation of Group C (low taskwork potential profile, high teamwork potential profile) = 0.597

Mean of Group D (low taskwork potential profile, low teamwork potential profile) = 4.211
Standard deviation of Group D (low taskwork potential profile, low teamwork potential profile) = 0.630

Levene's test for homogeneity of variances:
F-statistic = 4.227
df1 = 3
df2 = 253
p value= .006

ANOVA results for team taskwork potential profile:
F-statistic (degrees of freedom = 1,105) = 6.172
p-value = .013
Alpha level = .05
partial eta-squared (η^2) = .019

ANOVA results for team teamwork potential profile:
F-statistic (degrees of freedom = 1,105) = 9.596
p-value = .002
Alpha level = .05
partial eta-squared (η^2) = .029

ANOVA results for team taskwork potential profile & team teamwork potential profile interaction:
F-statistic (degrees of freedom = 1,105) = 0.374
p-value = .541
Alpha level = .05
partial eta-squared (η^2) = .001

Individual Profiles: ASI Advisor Dependable Ratings

Relevant data file:
SupplementaryData_IndividualPlayerProfiles_ASIonly

Analysis design:
Independent variable 1 = individual taskwork potential profile (low potential, high potential)
Independent variable 2 = individual teamwork potential profile (low potential, high potential)
Dependent variable = player ratings of whether advisor was dependable

Test type: two-way analysis of variance

Descriptive statistics:
Mean of Group A (high taskwork potential, high teamwork potential profile) = 4.645
Standard deviation of Group A (high taskwork potential profile, high teamwork potential profile)= 1.847

Mean of Group B (high taskwork potential profile, low teamwork potential profile)= 4.735
Standard deviation of Group B (high taskwork potential profile, low teamwork potential profile)= 1.868

Mean of Group C (low taskwork potential profile, high teamwork potential profile)= 4.793
Standard deviation of Group C (low taskwork potential profile, high teamwork potential profile) = 1.910

Mean of Group D (low taskwork potential profile, low teamwork potential profile) = 4.203
Standard deviation of Group D (low taskwork potential profile, low teamwork potential profile) = 1.747

Levene's test for homogeneity of variances:
F-statistic = 1.251
df1 = 3
df2 = 253
p value= .292

ANOVA results for team taskwork potential profile:
F-statistic (degrees of freedom = 1,253) = .671
p-value = .413
Alpha level = .05
partial eta-squared (η^2) = .003

ANOVA results for team teamwork potential profile:
F-statistic (degrees of freedom = 1,253) = 1.138
p-value = .287
Alpha level = .05
partial eta-squared (η^2) = .004

ANOVA results for team taskwork potential profile & team teamwork potential profile interaction:
F-statistic (degrees of freedom = 1,253) =2.099
p-value = .149
Alpha level = .05
partial eta-squared (η^2) = .008

Individual Profiles: Advisor Reasonable Ratings

Relevant data file:
SupplementaryData_IndividualPlayerProfiles_ASIonly

Analysis design:
Independent variable 1 = individual taskwork potential profile (low potential, high potential)
Independent variable 2 = individual teamwork potential profile (low potential, high potential)
Dependent variable = advisor rating: suggestions reasonable

Descriptive statistics:
Mean of Group A (high taskwork potential, high teamwork potential profile) = 5.23
Standard deviation of Group A (high taskwork potential profile, high teamwork potential profile)=1.641

Mean of Group B (high taskwork potential profile, low teamwork potential profile)= 5.673
Standard deviation of Group B (high taskwork potential profile, low teamwork potential profile)= 1.533

Mean of Group C (low taskwork potential profile, high teamwork potential profile)= 5.695
Standard deviation of Group C (low taskwork potential profile, high teamwork potential profile) = 1.385

Mean of Group D (low taskwork potential profile, low teamwork potential profile) = 4.969
Standard deviation of Group D (low taskwork potential profile, low teamwork potential profile) =1.759

Levene's test for homogeneity of variances:
F-statistic = 1.475
df1 = 3
df2 = 253
p value= .222

ANOVA results for team taskwork potential profile:
F-statistic (degrees of freedom = 1,105) = 0.356
p-value = .551
Alpha level = .05
partial eta-squared (η^2) = .001

ANOVA results for team teamwork potential profile:
F-statistic (degrees of freedom = 1,105) = 0.497
p-value = 0.482
Alpha level = .05
partial eta-squared (η^2) = .002

ANOVA results for team taskwork potential profile & team teamwork potential profile interaction:
F-statistic (degrees of freedom = 1,105) = 8.552
p-value = .004
Alpha level = .05
partial eta-squared (η^2) = .033

Team Profile Analysis Details

Team Profiles: Team taskwork-teamwork metric 1 (mission score %)

Relevant data file:
SupplementaryData_TeamProfiles

Analysis design:
Independent variable 1 = team taskwork potential profile (low potential, high potential)
Independent variable 2 = team teamwork potential profile (low potential, high potential)
Dependent variable = mission score percentage

Descriptive statistics:
Mean of Group A (high taskwork potential, high teamwork potential profile) = 69.368
Standard deviation of Group A (high taskwork potential profile, high teamwork potential profile)= 16.086

Mean of Group B (high taskwork potential profile, low teamwork potential profile)= 63.801
Standard deviation of Group B (high taskwork potential profile, low teamwork potential profile)= 12.361

Mean of Group C (low taskwork potential profile, high teamwork potential profile)= 56.096
Standard deviation of Group C (low taskwork potential profile, high teamwork potential profile) = 15.783

Mean of Group D (low taskwork potential profile, low teamwork potential profile) = 65.675
Standard deviation of Group D (low taskwork potential profile, low teamwork potential profile) = 12.571

Levene's test for homogeneity of variances:
F-statistic = 1.130
df1 = 3
df2 = 91

p value= .341

ANOVA results for team teamwork potential profile:
F-statistic (degrees of freedom = 1,91) =0.436
p-value = 0.511
Alpha level = .05
partial eta-squared (η^2) = .005

ANOVA results for team taskwork potential profile:
F-statistic (degrees of freedom = 1,91) = 3.518
p-value = 0.064
Alpha level = .05
partial eta-squared (η^2) = .037

ANOVA results for team teamwork potential profile & team taskwork potential profile interaction:
F-statistic (degrees of freedom = 1,91) = 6.212
p-value = .014
Alpha level = .05
partial eta-squared (η^2) = .064

Team profiles: taskwork-teamwork metric 2 (heal:extract)

Relevant data file:
SupplementaryData_Teamprofiles

Analysis design:
Independent variable 1 = team taskwork potential profile (low potential, high potential)
Independent variable 2 = team teamwork potential profile (low potential, high potential)
Dependent variable = team's heal-to-extract error rate

Descriptive statistics:
Mean of Group A (high taskwork potential, high teamwork potential profile) = 0.194
Standard deviation of Group A (high taskwork potential profile, high teamwork potential profile)= 0.119

Mean of Group B (high taskwork potential profile, low teamwork potential profile)= 0.267
Standard deviation of Group B (high taskwork potential profile, low teamwork potential profile)= 0.128

Mean of Group C (low taskwork potential profile, high teamwork potential profile)= 0.253
Standard deviation of Group C (low taskwork potential profile, high teamwork potential profile) = 0.143

Mean of Group D (low taskwork potential profile, low teamwork potential profile) = 0.216
Standard deviation of Group D (low taskwork potential profile, low teamwork potential profile) = 0.130

Levene's test for homogeneity of variances:
F-statistic = 0.423
df1 = 3
df2 = 91
p value= .737

ANOVA results for team taskwork potential profile:
F-statistic (degrees of freedom = 1,91) =.025
p-value = .875
Alpha level = .05
partial eta-squared (η^2) = .0002

ANOVA results for team teamwork potential profile:
F-statistic (degrees of freedom = 1,91) = .473
p-value = .493
Alpha level = .05
partial eta-squared (η²) =.005

ANOVA results for team taskwork potential profile & team teamwork potential profile interaction:
F-statistic (degrees of freedom = 1,91) = 4.120
p-value = .045
Alpha level = .05
partial eta-squared (η²) = .043

Team profiles: team taskwork-teamwork metric 3 (risk management error cost)

Relevant data file:
SupplementaryData_TeamProfiles

Analysis design:
Independent variable 1 = team taskwork potential profile (low potential, high potential)
Independent variable 2 = team teamwork potential profile (low potential, high potential)
Dependent variable = team's time cost for risk management errors

Descriptive statistics:
Mean of Group A (high taskwork potential, high teamwork potential profile) = 202749.759
Standard deviation of Group A (high taskwork potential profile, high teamwork potential profile)= 67214.989

Mean of Group B (high taskwork potential profile, low teamwork potential profile)= 281527.389
Standard deviation of Group B (high taskwork potential profile, low teamwork potential profile)= 127907.141

Mean of Group C (low taskwork potential profile, high teamwork potential profile)= 289662.0
Standard deviation of Group C (low taskwork potential profile, high teamwork potential profile) = 169480.146

Mean of Group D (low taskwork potential profile, low teamwork potential profile) = 219368.870
Standard deviation of Group D (low taskwork potential profile, low teamwork potential profile) = 107678.771

Levene's test for homogeneity of variances:
F-statistic = 3.734
df1 = 3
df2 = 90
p value= 0.014

ANOVA results for team taskwork potential profile:
F-statistic (degrees of freedom = 1,90) = 0.238
p-value = 0.627
Alpha level = .05
partial eta-squared (η²) = .003

ANOVA results for team teamwork potential profile:
F-statistic (degrees of freedom = 1,90) =0.028
p-value = 0.867
Alpha level = .05
partial eta-squared (η²) = .0003

ANOVA results for team taskwork potential profile & team teamwork potential profile interaction:
F-statistic (degrees of freedom = 1,90) = 8.647
p-value = .004
Alpha level = .05
partial eta-squared (η^2) = .088

Team profiles: team process perception

Relevant data file:
SupplementaryData_TeamProfiles_ASIonly

Analysis design:
Independent variable 1 = team taskwork potential profile (low potential, high potential)
Independent variable 2 = team teamwork potential profile (low potential, high potential)
Dependent variable = team's average team process rating

Descriptive statistics:
Mean of Group A (high taskwork potential, high teamwork potential profile) = 4.566
Standard deviation of Group A (high taskwork potential profile, high teamwork potential profile)= 0.248

Mean of Group B (high taskwork potential profile, low teamwork potential profile)= 4.306
Standard deviation of Group B (high taskwork potential profile, low teamwork potential profile)= 0.279

Mean of Group C (low taskwork potential profile, high teamwork potential profile)= 4.288
Standard deviation of Group C (low taskwork potential profile, high teamwork potential profile) = 0.573

Mean of Group D (low taskwork potential profile, low teamwork potential profile) = 4.464
Standard deviation of Group D (low taskwork potential profile, low teamwork potential profile) = 0.304

Levene's test for homogeneity of variances:
F-statistic = 7.136
df1 = 3
df2 = 63
p value= <.001

ANOVA results for team taskwork potential profile:
F-statistic (degrees of freedom = 1,63) = .015
p-value = 0.902
Alpha level = .05
partial eta-squared (η^2) =.0002

ANOVA results for team teamwork potential profile:
F-statistic (degrees of freedom = 1,63) = 0.834
p-value = .365
Alpha level = .05
partial eta-squared (η^2) = .013

ANOVA results for team taskwork potential profile & team teamwork potential profile interaction:
F-statistic (degrees of freedom = 1,63) = 6.340
p-value = .014
Alpha level = .05
partial eta-squared (η^2) = .091

Team profiles: ASI dependable

Relevant data file:
SupplementaryData_TeamProfiles_ASIonly

Analysis design:
Independent variable 1 = team taskwork potential profile (low potential, high potential)
Independent variable 2 = team teamwork potential profile (low potential, high potential)
Dependent variable = team's rating of whether ASI was dependable

Descriptive statistics:
Mean of Group A (high taskwork potential, high teamwork potential profile) = 4.284
Standard deviation of Group A (high taskwork potential profile, high teamwork potential profile)= 1.471

Mean of Group B (high taskwork potential profile, low teamwork potential profile)= 4.417
Standard deviation of Group B (high taskwork potential profile, low teamwork potential profile)= 1.111

Mean of Group C (low taskwork potential profile, high teamwork potential profile)= 4.651
Standard deviation of Group C (low taskwork potential profile, high teamwork potential profile) = 1.284

Mean of Group D (low taskwork potential profile, low teamwork potential profile) = 3.912
Standard deviation of Group D (low taskwork potential profile, low teamwork potential profile) = 0.936

Levene's test for homogeneity of variances:
F-statistic = 1.823
df1 = 3
df2 = 63
p value= .152

ANOVA results for team taskwork potential profile:
F-statistic (degrees of freedom = 1,63) = 0.051
p-value = .822
Alpha level = .05
partial eta-squared (η^2) = .0008

ANOVA results for team teamwork potential profile:
F-statistic (degrees of freedom = 1,63) = 0.980
p-value = .326
Alpha level = .05
partial eta-squared (η^2) = .015

ANOVA results for team taskwork potential profile & team teamwork potential profile interaction:
F-statistic (degrees of freedom = 1,63) = 2.022
p-value = .160
Alpha level = .05
partial eta-squared (η^2) = .031

Team profiles: ASI reasonable

Relevant data file:
SupplementaryData_TeamProfiles_ASIonly

Analysis design:
Independent variable 1 = team taskwork potential profile (low potential, high potential)
Independent variable 2 = team teamwork potential profile (low potential, high potential)

Dependent variable = team's rating of whether ASI was reasonable

Descriptive statistics:
Mean of Group A (high taskwork potential, high teamwork potential profile) = 4.804
Standard deviation of Group A (high taskwork potential profile, high teamwork potential profile)= 1.533

Mean of Group B (high taskwork potential profile, low teamwork potential profile)= 5.861
Standard deviation of Group B (high taskwork potential profile, low teamwork potential profile)= 0.926

Mean of Group C (low taskwork potential profile, high teamwork potential profile)= 5.349
Standard deviation of Group C (low taskwork potential profile, high teamwork potential profile) = 0.920

Mean of Group D (low taskwork potential profile, low teamwork potential profile) = 4.951
Standard deviation of Group D (low taskwork potential profile, low teamwork potential profile) = 0.935

Levene's test for homogeneity of variances:
F-statistic = 3.522
df1 = 3
df2 = 63
p value= .020

ANOVA results for team taskwork potential profile:
F-statistic (degrees of freedom = 1,63) = 0.433
p-value = .513
Alpha level = .05
partial eta-squared (η^2) = .007

ANOVA results for team teamwork potential profile:
F-statistic (degrees of freedom = 1,63) = 1.411
p-value = .239
Alpha level = .05
partial eta-squared (η^2) = .022

ANOVA results for team taskwork potential profile & team teamwork potential profile interaction:
F-statistic (degrees of freedom = 1,63) = 6.883
p-value = .011
Alpha level = .05
partial eta-squared (η^2) = 0.098

'Extreme' Team Profiles X Advisor Type: taskwork-teamwork metric 1 (mission score %)

Relevant data file:
SupplementaryData_ExtremeTeamProfiles

Analysis design:
Independent variable 1 = team holistic profile (low taskwork low teamwork, high taskwork high teamwork)
Independent variable 2 = advisor type (no advisor, human advisor, ASI advisor)
Dependent variable = team's mission score percentage

Descriptive statistics:
Mean of Group A (ASI advisor, high teamwork high taskwork) = 61.858
Standard deviation of Group A (ASI advisor, high teamwork high taskwork)= 16.918

Mean of Group B (ASI advisor, low teamwork low taskwork) = 67.554
Standard deviation of Group B (ASI advisor, low teamwork low taskwork)= 13.185

Mean of Group C (Human advisor, high teamwork high taskwork) = 79.368
Standard deviation of Group C (Human advisor, high teamwork high taskwork)= 2.844

Mean of Group D (Human advisor, low teamwork low taskwork) = 62.895
Standard deviation of Group D (Human advisor, low teamwork low taskwork)= 11.284

Mean of Group E (No advisor, high teamwork high taskwork) = 79.079
Standard deviation of Group E (No advisor, high teamwork high taskwork) = 9.849

Mean of Group F (No advisor, low teamwork low taskwork) = 55.263
Standard deviation of Group E (No advisor, low teamwork low taskwork) = 2.233

Levene's test for homogeneity of variances:
F-statistic = 3.262
df1 = 5
df2 = 47
p value= .013

ANOVA results for advisor type:
F-statistic (degrees of freedom = 2,47) = .828
p-value = .443
Alpha level = .05
partial eta-squared (η^2) = .034

ANOVA results for team profile:
F-statistic (degrees of freedom = 1,47) = 5.574
p-value = .022
Alpha level = .05
partial eta-squared (η^2) =.106

ANOVA results for profile X advisor type interaction:
F-statistic (degrees of freedom = 2,47) = 4.807
p-value = .013
Alpha level = .05
partial eta-squared (η^2) = .170

'Extreme' Team Profiles X Advisor Type: taskwork-teamwork metric 2 (risk management failure cost)

Relevant data file:
SupplementaryData_ExtremeTeamProfiles

Analysis design:
Independent variable 1 = team holistic profile (low taskwork low teamwork, high taskwork high teamwork)
Independent variable 2 = advisor type (no advisor, human advisor, ASI advisor)
Dependent variable = risk management failure cost [ms]

Descriptive statistics:
Mean of Group A (ASI advisor, high teamwork high taskwork) = 190467.188

Standard deviation of Group A (ASI advisor, high teamwork high taskwork)= 63597.949

Mean of Group B (ASI advisor, low teamwork low taskwork) = 202606.882
Standard deviation of Group B (ASI advisor, low teamwork low taskwork)= 98504.867

Mean of Group C (Human advisor, high teamwork high taskwork) = 185043.4
Standard deviation of Group C (Human advisor, high teamwork high taskwork)= 83386.464

Mean of Group D (Human advisor, low teamwork low taskwork) = 204497
Standard deviation of Group D (Human advisor, low teamwork low taskwork)= 72754.624

Mean of Group E (No advisor, high teamwork high taskwork) = 238381.375
Standard deviation of Group E (No advisor, high teamwork high taskwork) = 65627.625

Mean of Group F (No advisor, low teamwork low taskwork) = 391589.5
Standard deviation of Group E (No advisor, low teamwork low taskwork) = 137738.037

Levene's test for homogeneity of variances:
F-statistic = 0.635
df1 = 5
df2 = 46
p value= .674

ANOVA results for advisor type:
F-statistic (degrees of freedom = 2,47) = 5.962
p-value = .005
Alpha level = .05
partial eta-squared (η^2) = .206

ANOVA results for team profile:
F-statistic (degrees of freedom = 1,47) = 5.962
p-value = .043
Alpha level = .05
partial eta-squared (η^2) =.086

ANOVA results for profile X advisor type interaction:
F-statistic (degrees of freedom = 2,47) = 2.061
p-value = .139
Alpha level = .05
partial eta-squared (η^2) = .082

'Extreme' Team Profiles X Advisor Type: teamwork metric 1 (knowledge externalization)

Relevant data file:
SupplementaryData_ExtremeTeamProfiles

Analysis design:
Independent variable 1 = team holistic profile (low taskwork low teamwork, high taskwork high teamwork)
Independent variable 2 = advisor type (no advisor, human advisor, ASI advisor)
Dependent variable = team's usage of knowledge externalization tools

Descriptive statistics:
Mean of Group A (ASI advisor, high teamwork high taskwork) = 81.588

Standard deviation of Group A (ASI advisor, high teamwork high taskwork)= 23.773

Mean of Group B (ASI advisor, low teamwork low taskwork) = 109.059
Standard deviation of Group B (ASI advisor, low teamwork low taskwork)= 32.7

Mean of Group C (Human advisor, high teamwork high taskwork) = 107
Standard deviation of Group C (Human advisor, high teamwork high taskwork)= 37.855

Mean of Group D (Human advisor, low teamwork low taskwork) = 97.250
Standard deviation of Group D (Human advisor, low teamwork low taskwork)= 34.131

Mean of Group E (No advisor, high teamwork high taskwork) = 114.750
Standard deviation of Group E (No advisor, high teamwork high taskwork) = 39.008

Mean of Group F (No advisor, low teamwork low taskwork) = 26
Standard deviation of Group E (No advisor, low teamwork low taskwork) = 2.828

Levene's test for homogeneity of variances:
F-statistic = 0.825
df1 = 5
df2 = 47
p value= 0.538

ANOVA results for advisor type:
F-statistic (degrees of freedom = 2,47) = 2.141
p-value = 0.129
Alpha level = .05
partial eta-squared (η^2) = .084

ANOVA results for team profile:
F-statistic (degrees of freedom = 1,47) = 4.319
p-value = .043
Alpha level = .05
partial eta-squared (η^2) = .084

ANOVA results for team profile X advisor type:
F-statistic (degrees of freedom = 2,47) = 9.613
p-value = <.001
Alpha level = .05
partial eta-squared (η^2) = .290
